# Highly Selective Electrocatalytic Reduction of Substituted Nitrobenzenes to Their Aniline Derivatives Using a Polyoxometalate Redox Mediator

**DOI:** 10.1021/acsorginorgau.2c00047

**Published:** 2022-11-21

**Authors:** Athanasios D. Stergiou, Daniel H. Broadhurst, Mark D. Symes

**Affiliations:** WestCHEM, School of Chemistry, University of Glasgow, University Avenue, GlasgowG12 8QQ, U.K.

**Keywords:** nitroarene, aniline, electrocatalysis, redox mediator, polyoxometalate

## Abstract

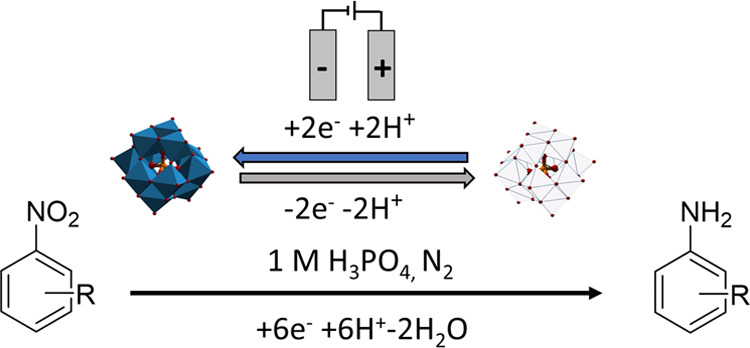

Anilines and substituted anilines are used on the multi-ton scale for producing polymers, pharmaceuticals, dyes, and other important compounds. Typically, these anilines are produced from their corresponding nitrobenzene precursors by reaction with hydrogen at high temperatures. However, this route suffers from a number of drawbacks, including the requirement to handle hydrogen gas, rather harsh reaction conditions that lead to a lack of selectivity and/or toleration of certain functional groups, and questionable environmental sustainability. In light of this, routes to the reduction of nitrobenzenes to their aniline derivatives that operate at room temperature, in aqueous solvent, and without the requirement to use harsh process conditions, hydrogen gas, or sacrificial reagents could be of tremendous benefit. Herein, we report on a highly selective electrocatalytic route for the reduction of nitrobenzenes to their corresponding anilines that works in aqueous solution at room temperature and which does not require the use of hydrogen gas or sacrificial reagents. The method uses a polyoxometalate redox mediator, which reversibly accepts electrons from the cathode and reacts with the nitrobenzenes in solution to reduce them to the corresponding anilines. A variety of substituted nitroarenes are explored as substrates, including those with potentially competing reducible groups and substrates that are difficult to reduce selectively by other means. In all cases, the selectivity for the redox-mediated route is higher than that for the direct reduction of the nitroarene substrates at the electrode, suggesting that redox-mediated electrochemical nitroarene reduction is a promising avenue for the more sustainable synthesis of substituted anilines.

## 1 Introduction

Aromatic amines (anilines) are key nitrogen-containing building blocks for the production of pharmaceuticals, dyes, and polymers, with a prime example being the synthesis of paracetamol, a derivative of 4-aminophenol.^1−3^ Typically, aromatic amines are synthesized through the reduction of the corresponding nitroarene compounds using heterogeneous catalysts at elevated temperatures and pressures and employing hydrogen gas as the reductant.^4,5^ Although these conditions give good results for the reduction of unfunctionalized nitrobenzene to aniline, the reduction of more complex (substituted) nitroarenes to their aniline products is much more difficult to achieve with high selectivity.^5,6^ From among the substituted nitroarenes with reducible groups (e.g., carbonyls, esters, amines, and halide groups), the most challenging substrates are the halogenated aromatics, as the hydro-dehalogenation reaction can occur very readily under the standard high temperature and pressure reduction conditions, leading to a marked drop in selectivity.^5−9^

In recent years, new methods have been developed to tackle the issue of the lack of chemoselectivity for the reduction of substituted nitroarenes. These methods have mostly focused on the replacement of H_2_ gas by a different hydrogenation agent (such as sodium borohydride, formate, or hydrazine) and/or on avoiding the need for high temperature and pressure.^2,10^ However, the aforementioned alternative reducing agents are generally toxic and are irreversibly consumed during the reduction process,^11,12^ and there is still the need to employ a heterogeneous catalyst.

A far simpler and more sustainable alternative to these current approaches to the synthesis of substituted anilines could be the electrochemical reduction of the corresponding nitrobenzenes; however, studies have shown that the lack of chemoselectivity toward the desired product (even in the case of unsubstituted nitrobenzene itself) remains a drawback when direct electroreduction of nitroarenes at the cathode is attempted.^13,14^ In recent years, numerous electrochemical methods have been developed, often using modified cathodes.^15−24^ Of these studies, the Zhang group reported the highest conversion and selectivity toward the desired products. However, hydrogen evolution competes with nitroarene reduction, impairing the faradic efficiency.^15,16^

Against this backdrop, the use of polyoxometalates as electro-generated reducing agents for the more selective reduction of nitrobenzene and allied substituted nitroarenes to their corresponding anilines has gained traction, both when used as reagents in excess^25^ and when used in catalytic amounts as part of an electrocatalytic cycle.^26,27^ In the latter case, the polyoxometalate mediator was shown to shut down the direct electrochemical reduction of the nitroarenes at the cathode that would otherwise lead to undesirable side reactions. However, the scope of the reactions reported in this previous study was limited, as our aim was primarily to elucidate the mechanism of reaction for the electrochemical reduction of nitrobenzene to aniline. Herein, we significantly expand the scope of this approach to a variety of substituted nitroarenes, including potentially competing reducible groups and substrates that are difficult to reduce selectively by other means. In all cases, we compare the electro-mediated approach to the direct reduction of the nitroarene substrates at the electrode and find that the use of the polyoxometalate redox mediator almost always leads to better conversions of the starting material and always gives higher selectivity for the aniline product.

## 2 Experimental Section

### 2.1 General Experimental Remarks

1-bromo-4-nitrobenzene (98%), ethyl-4-nitrobenzoate (98%), 1-fluoro-2-nitrobenzene (99%), 2-nitrophenol (98%), 1-iodo-2-nitrobenzene (97%), 4-nitroacetophenone (98%), 1-fluoro-4-nitrobenzene (99%), 4-nitrobenzoic acid (99%), and 1-chloro-2-nitrobenzene (99%) were purchased from Alfa Aesar. 4-nitroanisole (97%), methyl-2-nitrobenzoate (>98%), and 2-nitrobenzonitrile (>98%) were purchased from Sigma-Aldrich while ethyl-3-nitrobenzoate (99%) was purchased from Fluorochem. Phosphotungstic acid (reagent grade) was supplied by both Sigma-Aldrich and Alfa Aesar. Silicotungstic acid (reagent grade) was purchased from Sigma-Aldrich. Chloroform (99.8%), acetone (99.5%), and phosphoric acid (85%) were purchased from Fisher Scientific. Dichloromethane (99.5%) and magnesium sulfate (99.2%) were purchased from VWR, while diethyl ether (99.5%) was purchased from Scientific Laboratory Supplies Ltd. Deuterated chloroform (99.8%), dimethyl sulfoxide (99.9%), and methanol (99.8%) were supplied by Cambridge Isotope Laboratories. 254 μm-thick Nafion N-1110 membrane, used in the H-cells, was purchased from Fuel Cell Store and soaked in 1 M sulfuric acid solution overnight prior to use. Otherwise, all chemical reagents and solvents were used as purchased. Carbon felt, used as a high-surface-area electrode, was purchased from Alfa Aesar (3.18 mm thick, 99.0%). All electrolyte solutions were prepared with ultrapure deionized water (18.2 MΩ-cm resistivity) obtained from a Sartorius Arium Comfort combined water system. All NMR data were collected using a Bruker AV 400 instrument at a constant temperature of 300 K. pH determinations were made with a Hanna HI 9025 waterproof pH meter. All other materials were obtained, as stated in the text. Experiments performed at “room temperature” were carried out at 25 °C.

### 2.2 General Electrochemical Methods

Electrochemical studies were performed in a three-electrode configuration (unless otherwise stated) using either a CH Instruments CHI600D potentiostat or a BioLogic SP-150 potentiostat. A glassy carbon button electrode (surface area = 0.071 cm^2^) or carbon felt was used as the working electrodes (as specified), a Pt wire or a piece of carbon felt was used as the counter electrode (as specified), and an Ag/AgCl (NaCl, 3 M) reference electrode was used when specified. Glassy carbon working electrodes were polished using polishing powder and then washed with acetone and deionized water prior to use. Carbon felt electrodes were not reused.

### 2.3 Cyclic Voltammetry

Cyclic voltammograms were collected in single chamber cells using a three-electrode setup at room temperature at a scan rate of 10 mV/s (unless otherwise stated) in 1 M aqueous H_3_PO_4_ electrolyte. The solvent (10 mL) was thoroughly degassed with N_2_ prior to the experiments and kept under an inert atmosphere throughout the process. Typically, to this degassed solvent was added 9.75 × 10^–5^ mol of the relevant nitroarene substrate (unless noted otherwise). To this solution, 1 equiv mol of H_3_[PW_12_O_40_] (0.28 g) was then added to the electrochemical cell, followed by degassing and stirring for 5 min. A glassy carbon button electrode was used as the working electrode (area = 0.071 cm^2^), a Pt wire was used as the counter electrode, and an Ag/AgCl (3 M NaCl) reference electrode was used. Measurements were conducted without stirring and with iR compensation enabled. The IUPAC convention was used when plotting the cyclic voltammograms.

### 2.4 Electrocatalytic Studies

Electrocatalytic studies were performed as follows. Unless otherwise stated, 9.74 × 10^–4^ mol of the relevant nitroarene was added to 30 mL of a 3.3 mM aqueous solution of phosphotungstic acid (i.e., a 10 mol % ratio of phosphotungstic acid relative to the nitroarene). This solution was then placed into the working electrode compartment of an H-cell. For the less soluble nitroarenes (ethyl-2-nitrobenzoate, 4-nitroacetophenone, etc.), half the amount of starting material was used, i.e., 4.87 × 10^–4^ mol, but still with a 10 mol % ratio of phosphotungstic acid (1.65 mM) relative to the nitroarene. The counter electrode side of the cell was filled with 1 M aqueous H_3_PO_4_ electrolyte solution. The two compartments of the H-cell were separated by a Nafion N-1110 membrane (see Figure S1 for a representation of the electrolysis setup). Typically, bulk electrolysis was then carried out at −0.38 V vs Ag/AgCl under an inert atmosphere until substrate reduction was complete, as judged by the falling off of the current to background levels. The working and counter electrodes for bulk electrolysis were both rectangular strips of carbon felt (of dimension 3 × 2 cm^2^), and an Ag/AgCl reference electrode was used.

After electrolysis for a given time, the (now dark blue) solution was removed from the working electrode compartment of the H-cell. The pH of this solution was then raised above the p*K*_a_ value of the anticipated product using 1 M NaOH to deprotonate the R-NH_3_^+^ salt and form the neutral R-NH_2_ state. This in turn allowed the reduced organic product to be extracted into organic solvents for isolation. The pH values in question for the various conversions were: 2-nitrophenol to 2-aminophenol, pH = 6.0, 1-bromo-4-nitrobenzene to 4-bromoaniline, pH = 6.1, 2-nitrotoluene to 2-aminotoluene, pH = 5.7, 2-nitrobenzonitrile to 2-aminobenzonitrile, pH = 6.1, methyl-2-nitrobenzoate to methyl-2-aminobenzoate, pH = 3.5, 1-chloro-2-nitrobenzene to 2-chloroaniline, pH = 3.7, ethyl-4-nitrobenzoate to ethyl-4-aminobenzoate, pH = 5.0, 4-nitrobenzoic acid to 4-aminobenzoic acid, pH = 3.7, 4-fluoro-1-nitrobenzene to 4-fluoroaniline, pH = 6.3, 4-nitroacetophenone to 4-aminoacetophenone, pH = 4.0, 1-iodo-3-nitrobenzene to 3-iodoaniline, pH = 5.0, and ethyl-3-nitrobenzoate to ethyl-3-aminobenzoate, pH = 4.0.

Ethyl-4-aminobenzoate, ethyl-3-aminobenzoate, and 3-iodoaniline were extracted using chloroform, 2-aminophenol was extracted using dichloromethane and 4-bromoaniline, 4-fluoroaniline, 2-aminotoluene, methyl-2-aminobenzoate, 2-aminobenzonitrile, 2-chloroaniline, 4-aminobenzoic acid, and 4-aminoacetophenone were extracted using diethyl ether. In all cases, after extraction, magnesium sulfate was added to the organic phase to remove any remaining water. The organic phase was then filtered and concentrated under reduced pressure using a rotary evaporator to give the isolated reduced aniline derivatives.

### 2.5 ^1^H NMR Determination of Conversions

After extraction into organic solvent and concentration under reduced pressure, the conversion of the starting material was determined *via* the integration of the relevant ^1^H NMR peaks. After the ^1^H NMR peaks in any given spectrum had been identified and assigned, peaks corresponding to the same number of protons in both the starting material and the various products were compared, allowing the percentage of starting material converted to each product (or not converted, and hence still present as starting material) to be determined.

### 2.6 Product Yields

The yields and masses (the latter in parentheses) of the compounds listed in Table 1 are as follows: 2-nitrophenol (Table 1, entry 1) 72% (77 mg); 4-bromo-1-nitrobenzene (Table 1, entry 2) 39% (35 mg); 2-nitrotoluene (Table 1, entry 3) 6% (8 mg); 2-nitrobenzonitrile (Table 1, entry 4) 61% (36 mg); methyl-2-nitrobenzoate (Table 1, entry 5) 63% (46 mg); 2-chloro-1-nitrobenzene (Table 1, entry 6) 45% (30 mg); ethyl-4-nitrobenzoate (Table 1, entry 7) 38% (49 mg); 4-nitrobenzoic acid (Table 1, entry 8) 33% (38 mg); 4-fluoro-1-nitrobenzene (Table 1, entry 9) 57% (31 mg); 4-nitroacetophenone (Table 1, entry 10) 67% (50 mg); 1-iodo-3-nitrobenzene (Table 1, entry 11) 42% (45 mg), and ethyl-3-nitrobenzoate (Table 1, entry 12) 52% (45 mg). The low isolated yields of some of these species are due to the comparatively small scale of the reaction and associated losses during the extraction process.

**Table 1:**
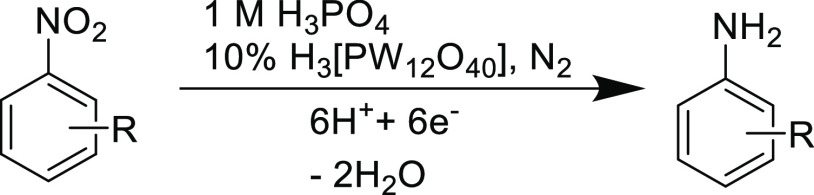

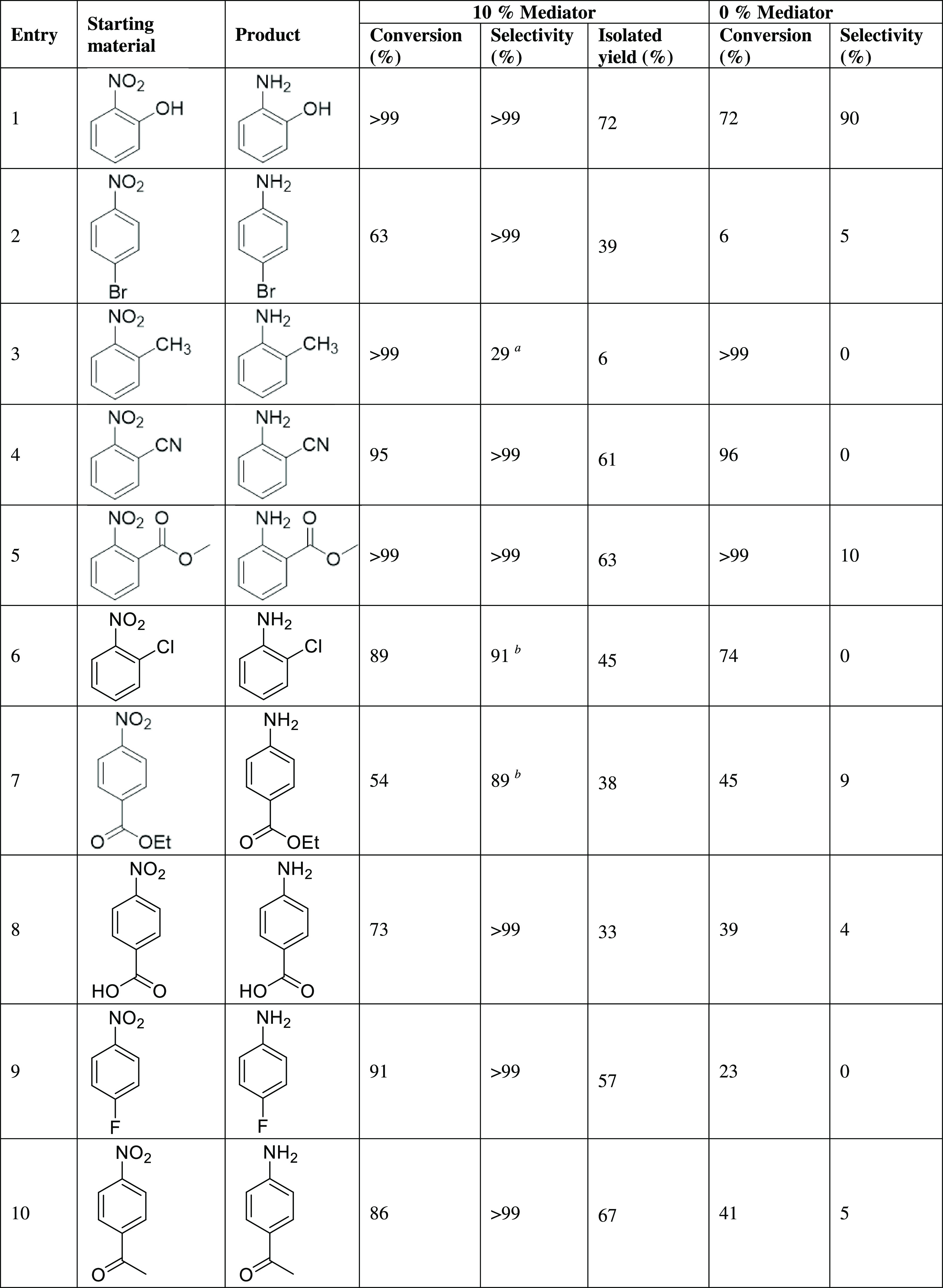

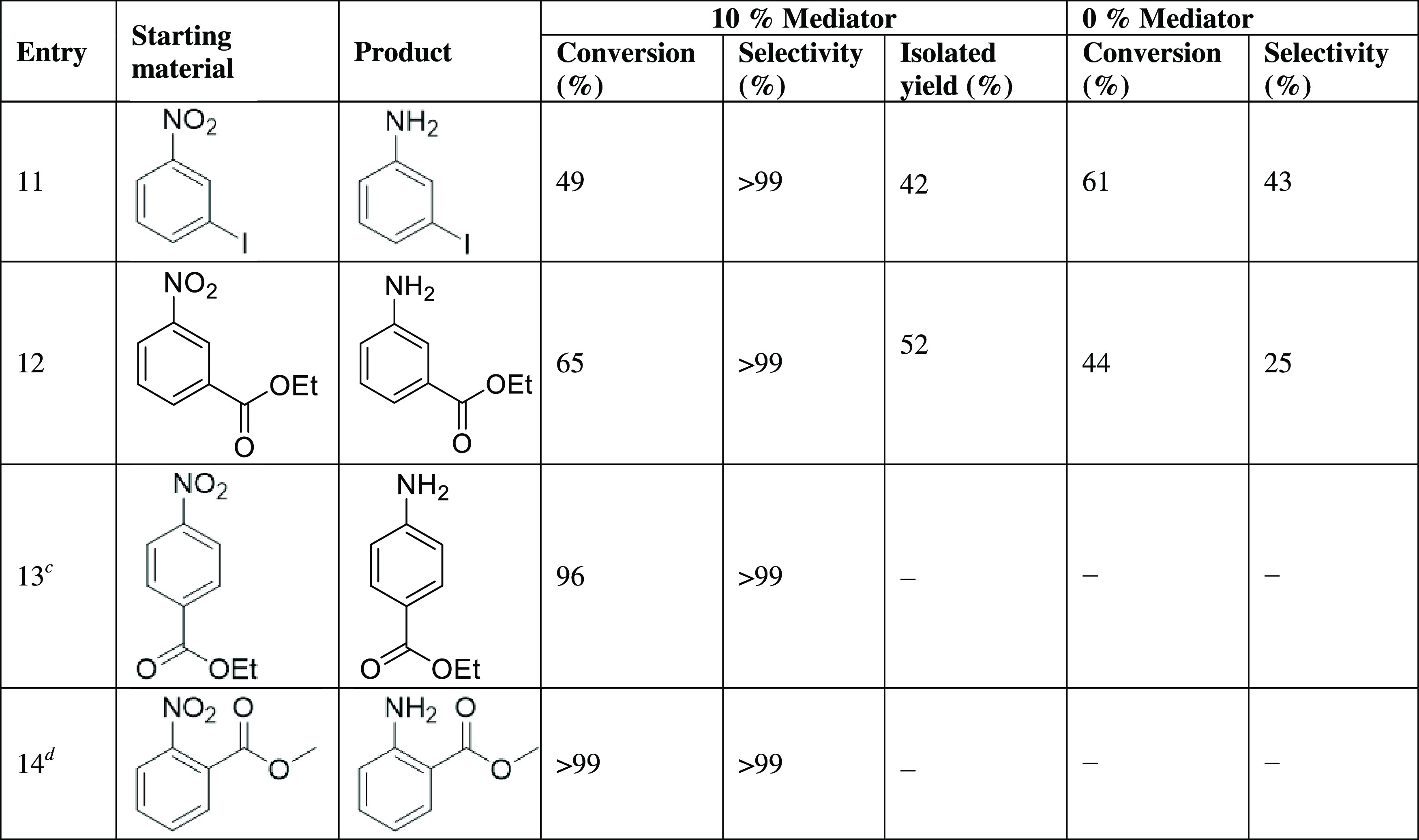
Comparison between the Mediated and Nonmediated Nitroarene Reduction Reactions

a The main product of this reaction is the hydroxylamine intermediate, as identified by ESI-mass spectrometry (*m*/*z* = 124.0760 for [M + H]^+^).

b The balance of the starting material of this reaction was found to have converted to the hydroxylamine intermediate, as identified by ^1^H NMR.

c This reaction was conducted at 50 °C.

d This reaction was performed using the redox mediator silicotungstic acid at −0.56 V vs Ag/AgCl, with the rest of the conditions being the same as for entry 5.

## 3 Results and Discussion

### 3.1 Voltammetry

**Figure 1:**
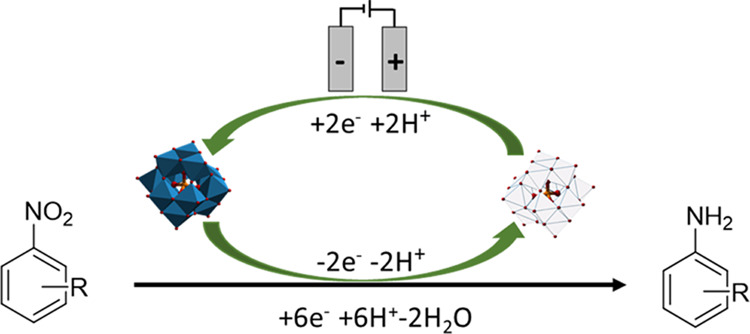
Illustration of the electrocatalytic hydrogenation of nitrobenzenes using a phosphotungstic acid redox mediator.

A diagrammatic representation of the redox-mediated approach^28^ for the reduction of nitroarenes is depicted below (Figure 1), where the polyoxometalate mediator is electrochemically reduced at the surface of the cathode and subsequently reacts in solution with the corresponding nitrobenzene to yield the aniline derivative. The mediator becomes reoxidized in the process and is thus able to be reduced again at the cathode (leading to turnover). The corresponding direct electrochemical reduction reactions (i.e., in the absence of any mediator) were also studied to provide a comprehensive understanding of the mediated reactions.

**Figure 2:**
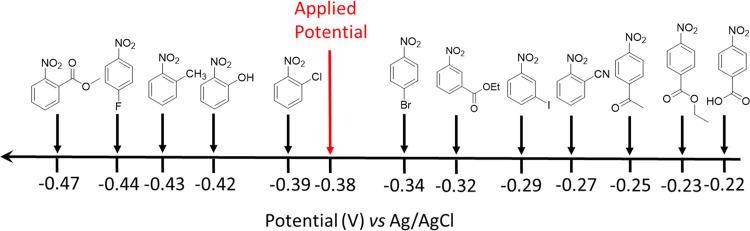
Schematic illustration of the reduction potentials of the examined substituted nitrobenzenes. The reduction potentials were extracted from the cyclic voltammograms and were recorded using a glassy carbon working electrode (surface area = 0.071 cm^2^), a Pt wire counter electrode, and an Ag/AgCl reference electrode. The electrolyte was 1 M aqueous H_3_PO_4_, and the scan rate was 10 mV/s in all cases. The scale bar is nonlinear to allow the relative ordering of the various redox processes to be more clearly seen.

In the following, the mediated experiments were performed using 10 mol % of the redox mediator relative to the starting material. Cyclic voltammograms of all of the selected substrates in 1 M H_3_PO_4_ were recorded (see Figures S2–S13), and the data obtained for the (peak) positions of the redox waves of these substrates relative to the peak position of the second reversible reduction wave of the polyoxometalate mediator (−0.38 V vs Ag/AgCl) are summarized in Figure 2. The polyoxometalate mediator is only stable at low pH, so only low-pH conditions were studied in this work.

**Figure 3:**
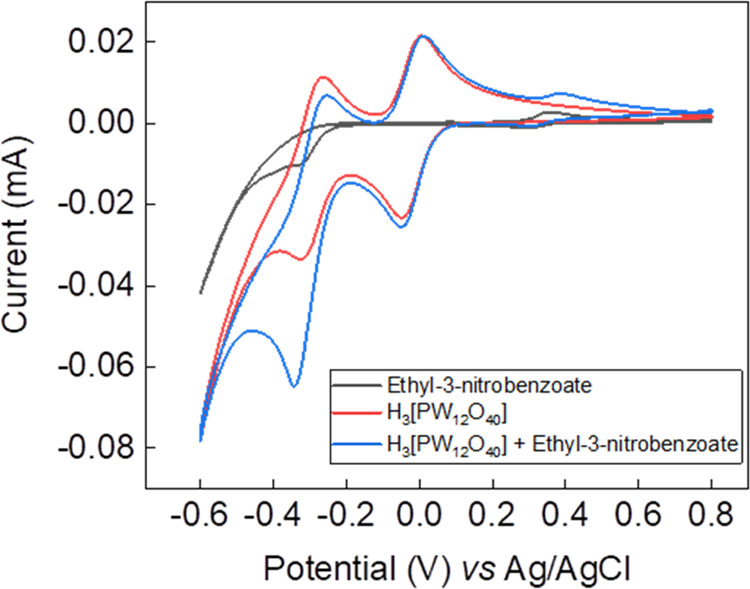
Cyclic voltammogram of ethyl-3-nitrobenzoate in 10 mL of 1 M aqueous H_3_PO_4_, using 9.74 × 10^–5^ mol of both the nitroarene and the redox mediator. A glassy carbon working electrode (surface area = 0.071 cm^2^), a Pt wire counter electrode, and an Ag/AgCl reference electrode were used. The scan rate was 10 mV/s.

An example of the reactivity of the redox mediator is depicted in Figure 3 for the starting material ethyl-3-nitrobenzoate. The red trace represents the electro-activity of the phosphotungstic acid redox mediator on its own, exhibiting two reversible one-electron redox waves at −0.04 and −0.32 V vs Ag/AgCl. The black trace represents the direct reduction of ethyl-3-nitrobenzoate at the electrode surface in the absence of the polyoxometalate mediator, with the main feature being an irreversible reduction at around −0.3 V vs Ag/AgCl. When the phosphotungstic acid redox mediator is present together with ethyl-3-nitrobenzoate, the second reduction peak of the redox mediator at −0.32 V vs Ag/AgCl exhibits enhanced reductive current, indicating that an electrocatalytic process is occurring between the two-electron reduced mediator and the substrate.

### 3.2 Substrate Scope

Table 1 summarizes the conversion of the nitroarene starting materials and the selectivity for the aniline derivatives for the various substrates listed under both mediated and direct electrochemical reduction (the latter corresponding to “0% mediator”). The electrolysis time of the mediated reaction (as judged by the fall of current to background levels, see Figure S26 for an example) varied from 7 to 11 h, with the less soluble substrates (for example, Table 1, entries 2 and 7) requiring longer reaction times. Six moles of electrons are required per mole of substrate for the complete transformation of substituted nitrobenzene to the corresponding aniline derivative. The faradic efficiency of the reaction, as calculated using Faraday’s law of electrolysis (eq 1) for each of the substrates in question, is listed below.
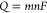
1where *Q* is the charge passed in C, *m* is the number of moles of the electroactive species in question, *n* is the number of electrons transferred per mole of electroactive species, and *F* is the Faraday constant and is equal to 96 485C mol^–1^. For conversion of nitrobenzene to aniline, *n* = 6.

The faradic yield for the formation of 2-aminophenol (Table 1, entry 1) was 68%, the faradic yield for the formation of 4-bromoaniline (Table 1, entry 2) was 40%, the faradic yield for the formation of 2-aminotoluene (Table 1, entry 3) was 14%, the faradic yield for the formation of 2-aminobenzonitrile (Table 1, entry 4) was 59%, the faradic yield for the formation of methyl-2-aminobenzoate (Table 1, entry 5) was 55%, the faradic yield for the formation of 2-chloroaniline (Table 1, entry 6) was 49%, the faradic yield for the formation of ethyl-4-aminobenzoate (Table 1, entry 7) was 45%, the faradic yield for the formation of 4-aminobenzoic acid (Table 1, entry 8) was 77%, the faradic yield for the formation of 4-fluoroaniline (Table 1, entry 9) was 52%, the faradic yield for the formation of 4-aminoacetophenone (Table 1, entry 10) was 78%, the faradic yield for the formation of 3-iodoanilne (Table 1, entry 11) was 68%, and the faradic yield for the formation of ethyl-3-aminobenzoate (Table 1, entry 12) was 61%. It should be noted that a thorough optimization of the faradic yields was not attempted, and hence it may be possible to improve these efficiencies with alterations to the reaction conditions.

As can be seen from Table 1, the use of the polyoxometalate redox mediator usually improves the conversion of the starting material and always improves the selectivity for conversion of the starting material to the aniline derivative when compared to the result found in the corresponding direct electrochemical reduction reaction. Figure 4 illustrates stacked ^1^H NMR plots for one such example, using methyl-2-nitrobenzoate as the substrate (Table 1, entry 5). ^1^H NMR stacked plots for the other substrates that were probed can be found in the Supporting Information (Figures S14–S25). Full range ^1^H NMR spectra of all isolated products can also be found in the Supporting Information (Figures S27–S38).

**Figure 4:**
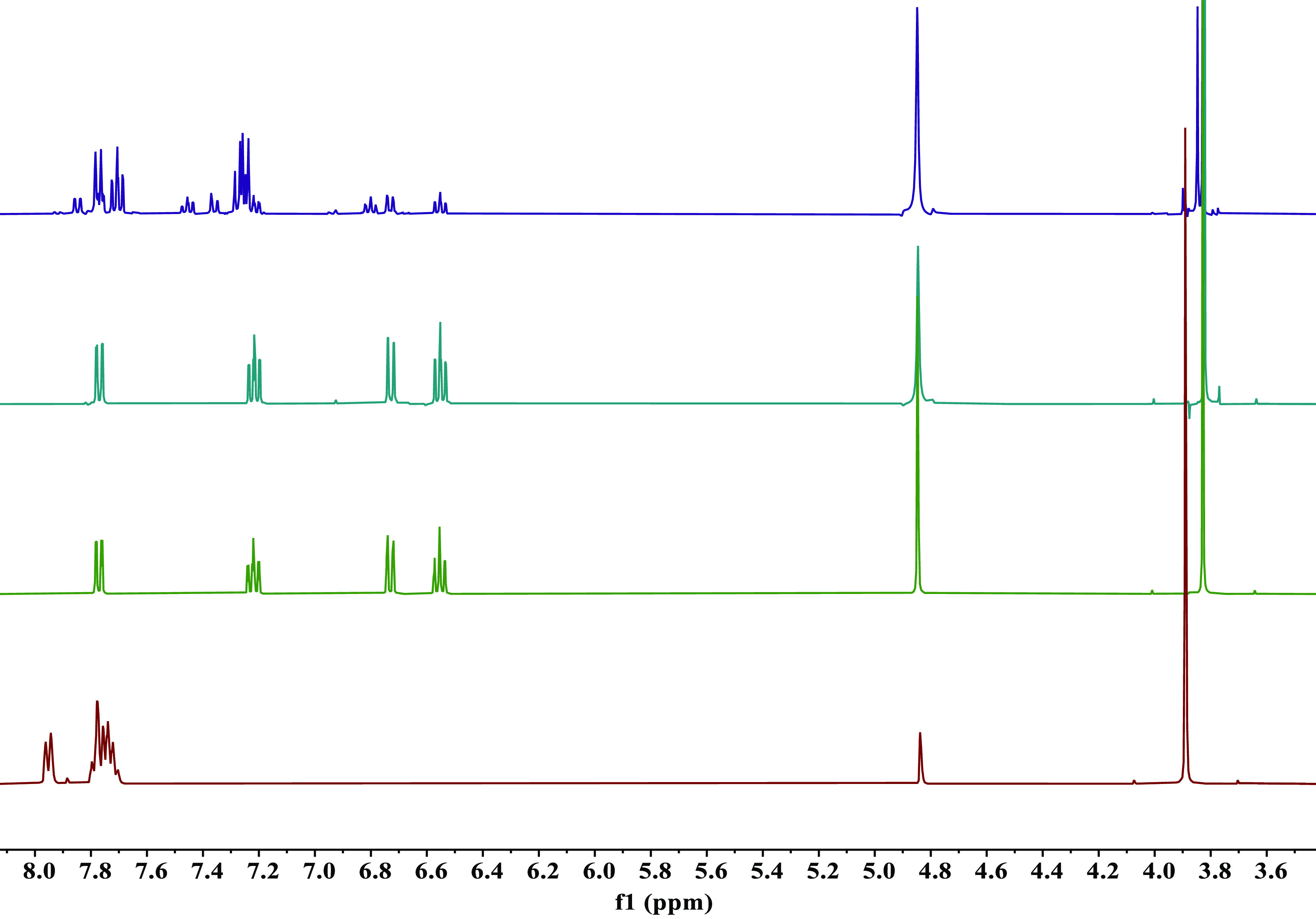
Stacked ^1^H NMR plot (MeOD, 300 K, 400 MHz) summarizing the outcome of the mediated electroreduction of methyl-2-nitrobenzoate. Shown in purple at the top is a spectrum of the extracted and concentrated reaction medium from a nonmediated reaction (direct substrate reduction at the cathode). Beneath this (turquoise) is a spectrum of the extracted and concentrated reaction medium from a mediated electrochemical reduction of methyl-2-nitrobenzoate. Beneath this (green) is a pure methyl-2-aminobenzoate standard sample. The bottom spectrum (red) is that of the starting material. The peak at 4.78 ppm originates from residual MeOH.

Even in the cases of halide-substituted nitrobenzenes (−F, −Cl, −Br, −I; Table 1, entries 2, 6, 9, and 11), which often suffer from cleavage of the halide from the aromatic ring under standard hydrogenation conditions, the selectivities for the production of the halide-substituted aniline compounds are very high. This is in contrast to the 0% mediator reaction outcomes under otherwise identical conditions, where both conversions and/or selectivities are generally markedly poorer. In some cases in Table 1, conversion of the starting material is less than quantitative (meaning in the case of the mediated reaction in entry 8, for example, that the reaction mixture contains just starting material and aniline product at the end of the reaction in a 27:73 ratio). Such instances of less than complete conversion are due to the low solubility of the relevant substrates in the aqueous electrolyte.

However, it is significant that the mediated reaction still proceeds highly selectively despite the poor solubility of some of the substrates, generally producing only the desired aniline derivative as the sole reduction product. The fact that such “on water” reactions are feasible with the mediated system enhances the sustainability aspects of this approach.

Reactions at elevated temperature (50 °C) were conducted using the starting material ethyl-4-nitrobenzoate (Table 1, entry 13), which showed sub-optimal conversion and selectivity when the reaction was conducted under otherwise identical conditions but at room temperature (Table 1, entry 7). The ^1^H NMR analysis of the reaction products from entry 13 showed that the selectivity of the reaction improved to >99%, and the conversion of starting material also improved from 54 to 96% when compared with the mediated electrolysis at room temperature (entry 7). Therefore, the use of elevated temperatures might aid the mediated reduction of substrates of poor solubility, provided issues with substrate/product/electrolyte evaporation and decomposition can be mitigated.

### 3.3 Scaled-Up Reactions

**Figure 5:**
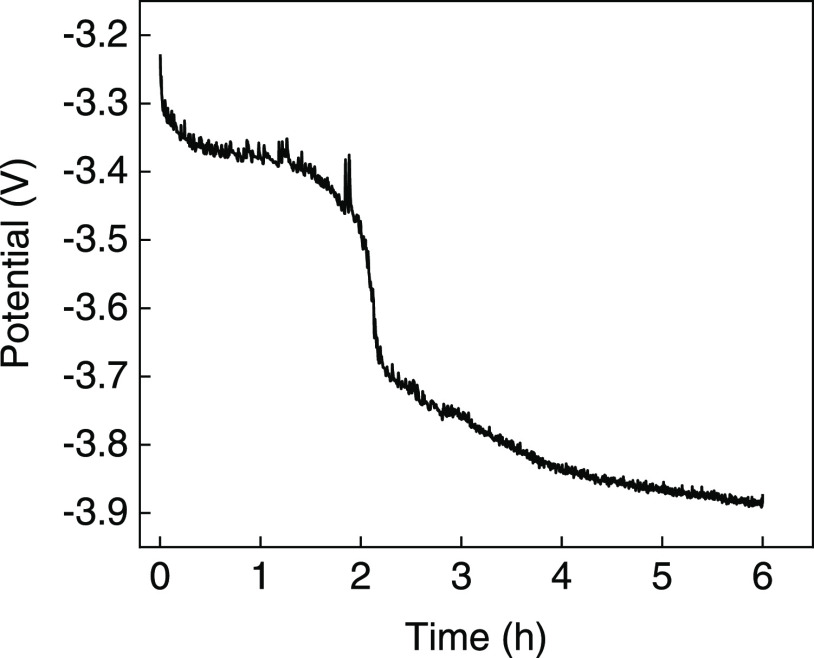
Voltage–time plot of the controlled current reduction of methyl-2-nitrobenzoate in 1 M aqueous H_3_PO_4_ at −20 mA using a 2-electrode setup. A platinum mesh was used as the counter electrode (anode), and carbon felt was used as the working electrode (cathode).

To show that there is the potential to develop this into a process for nitroarene reduction under preparative conditions (where the ability to operate at a larger scale, without the need for a reference electrode, and under controlled current would be useful), galvanostatic electrolysis on the substrate methyl-2-nitrobenzoate was performed using a two-electrode setup in an H-cell. All components were as described above, apart from the counter electrode, which was replaced with a large surface area Pt mesh. 90 mg of methyl-2-nitrobenzoate was thus dissolved in 35 mL of 1 M aqueous H_3_PO_4_ (giving a 14 mM solution) in the presence of 10% mol equiv of the redox mediator. The chosen applied cathodic current was 20 mA (current density = 2.7 mA/cm^2^). After a reaction time of 6 h, 92% of the starting material had been converted to the aniline product (2-aminobenzoic acid methyl ester) with >99% selectivity (i.e., no other reduction product was detected). Figure 5 shows a *V*–*t* curve for this reaction. A gram-scale reaction was also performed, maintaining the same current density but increasing the size of the electrochemical cell. Therefore, 1 g of starting material was dissolved in 100 mL of 1 M aqueous H_3_PO_4_ in the presence of 10% mol equiv of the redox mediator. After 22 h, the conversion of the in-solution substrate was 93%. However, some of the starting material had precipitated, meaning that the conversion was not quantitative and resulted in a total isolated yield of 2-aminobenzoic acid methyl ester of only 60% (0.41 g). The selectivity of the reaction for the material that did convert was still very high (>97%), meaning that essentially only the desired aminobenzoic acid methyl ester product and the starting material were present in the reaction mixture. The faradic yield was also impacted by the precipitation of the starting material, but even so, it was reasonable at 46%.

### 3.4 Silicotungstic Acid as an Alternative Mediator for Electrochemical Nitroarene Reduction

**Figure 6:**
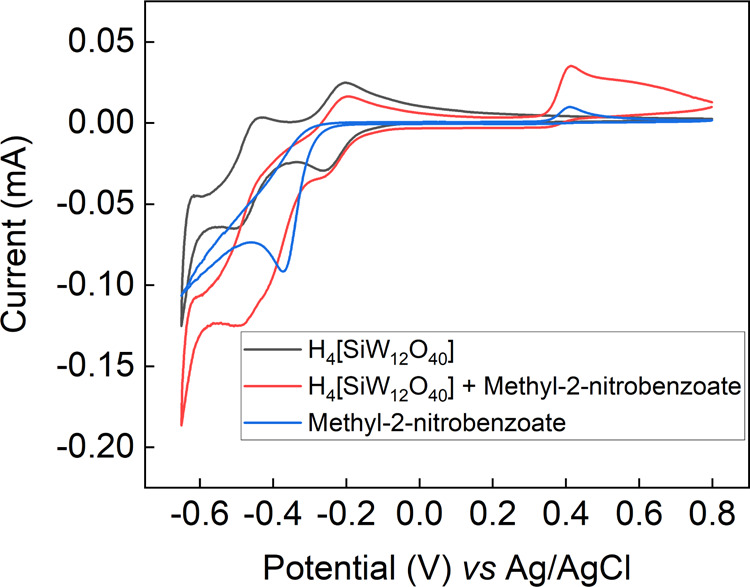
Cyclic voltammogram of methyl-2-nitrobenzoate in 10 mL 1 M aqueous H_3_PO_4_, using 9.74 × 10^–5^ mol of both the nitroarene and the redox mediator. A glassy carbon working electrode (surface area = 0.071 cm^2^), a Pt wire counter electrode, and an Ag/AgCl reference electrode were used. The scan rate was 10 mV/s.

To compare the electrocatalytic properties of alternative polyoxometalates as redox mediators for the electrochemical reduction of nitroarenes, the starting material methyl-2-nitrobenzoate (Table 1, entry 5) was selected, alongside silicotungstic acid (H_4_[SiW_12_O_40_]), as the redox mediator. Cyclic voltammetry using the same conditions as before (1 M aqueous H_3_PO_4_, 9.74 × 10^–5^ mol of both the nitroarene and the redox mediator) was performed, and the results are presented in Figure 6. The black trace represents the electro-activity of the silicotungstic acid redox mediator on its own, exhibiting two reversible one-electron redox waves at −0.26 and −0.48 V vs Ag/AgCl. The blue trace represents the direct reduction of methyl-2-nitrobenzoate at the electrode surface in the absence of the polyoxometalate mediator, with the main feature being an irreversible reduction at around −0.35 V vs Ag/AgCl. When the silicotungstic acid redox mediator is present together with methyl-2-nitrobenzoate, the second reduction peak of the redox mediator at −0.48 V vs Ag/AgCl exhibits enhanced reductive current, indicating again that in this case, an electrocatalytic process is occurring between the two-electron reduced mediator and the substrate.

Accordingly, bulk electrolysis was performed at −0.56 V vs Ag/AgCl (slightly cathodic of the second reduction wave of the mediator), with the rest of the conditions remaining the same as in Table 1, entry 5, i.e., 4.87 × 10^–4^ mol of starting material and 10 mol % of the redox mediator relative to the nitroarene. The ^1^H NMR analysis of (and electrochemical data for) the reaction using silicotungstic acid as the redox mediator (Table 1, entry 14) revealed a similar performance to the one reported for the phosphotungstic acid redox mediator (Table 1, entry 5), with the conversion and selectivity both found to be >99%. However, generating silicotungstic acid that is reduced by two electrons requires more cathodic potentials than those required to produce phosphotungstic acid that is reduced by two electrons (by roughly 150 mV). This in turn means that the required cell potentials are greater when using silicotungstic acid, leading to a higher energy demand for the reduction process using silicotungstic acid.

## 4 Conclusions

Herein, we have shown that polyoxometalate phosphotungstic acid is an effective redox mediator for the electrocatalytic reduction of halogenated, and alkyl-, carbonyl-, ester-, hydroxyl-, cyano-, and acid-substituted nitrobenzenes. In all cases, electrochemical reduction of these substrates in the presence of the redox mediator occurred with higher selectivity than for the direct reduction of the substrates at the electrode surface. Scaled-up reactions were also effective, returning only the desired aniline product and unreacted starting material, with essentially no formation of unwanted side-products. The process is notable not only for its high selectivity but also for its potential sustainability advantages over conventional methods for nitrobenzene reduction, including mild process conditions (aqueous solution at room temperature), the ability to work without extraneous reducing agents (including hydrogen gas), and the fact that a heterogeneous precious metal catalyst is not required. Further optimization of these procedures is currently underway in our laboratories.

## Data Availability

The data underpinning this study have been data-availability-deposited in the University of Glasgow’s Enlighten database under accession code http://dx.doi.org/10.5525/gla.researchdata.1365.

## Supporting Information Available

Cyclic voltammograms of all substrates, stacked ^1^H NMR spectra of all reaction outcomes, and ^1^H NMR spectra of purified reaction products (PDF)
