# Supplementary material for: Highly Selective Electrocatalytic Reduction of Substituted Nitrobenzenes to Their Aniline Derivatives Using a Polyoxometalate Redox Mediator
Source: ACS Org Inorg Au. 2022 Nov 21;3(1):51–8. doi: 10.1021/acsorginorgau.2c00047 (PMC9896480; doi:10.1021/acsorginorgau.2c00047)
Supplement: Supplementary file 1 — gg2c00047_si_001.pdf [file gg2c00047_si_001.pdf]

Supporting Information for:

Highly Selective Electrocatalytic Reduction of Substituted Nitrobenzenes to their Aniline Derivatives using a Polyoxometalate Redox Mediator

*Athanasios D. Stergiou, Daniel H. Broadhurst and Mark D. Symes**

WestCHEM, School of Chemistry, University of Glasgow, University Avenue, Glasgow, G12 8QQ, UK.

**E-mail: mark.symes@glasgow.ac.uk*

Electrolysis setup

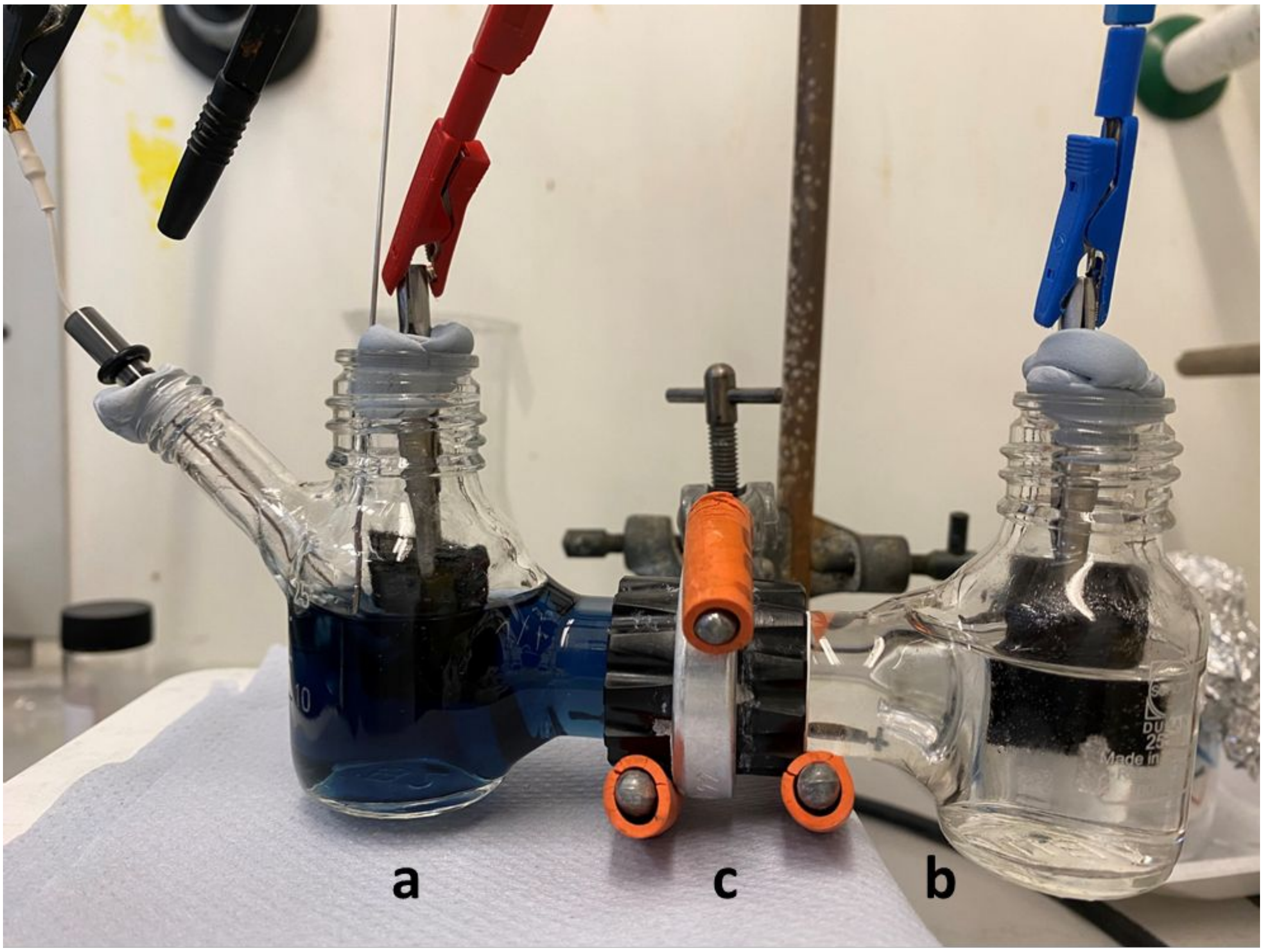

Figure S1: Image of the electrolysis set-up. a) working electrode side of the H-cell containing the working and reference electrode, the redox mediator and the starting material, b) counter electrode side of the H-cell containing the counter electrode and the electrolyte, and c) the Nafion membrane that separates the two compartments.

Cyclic Voltammograms

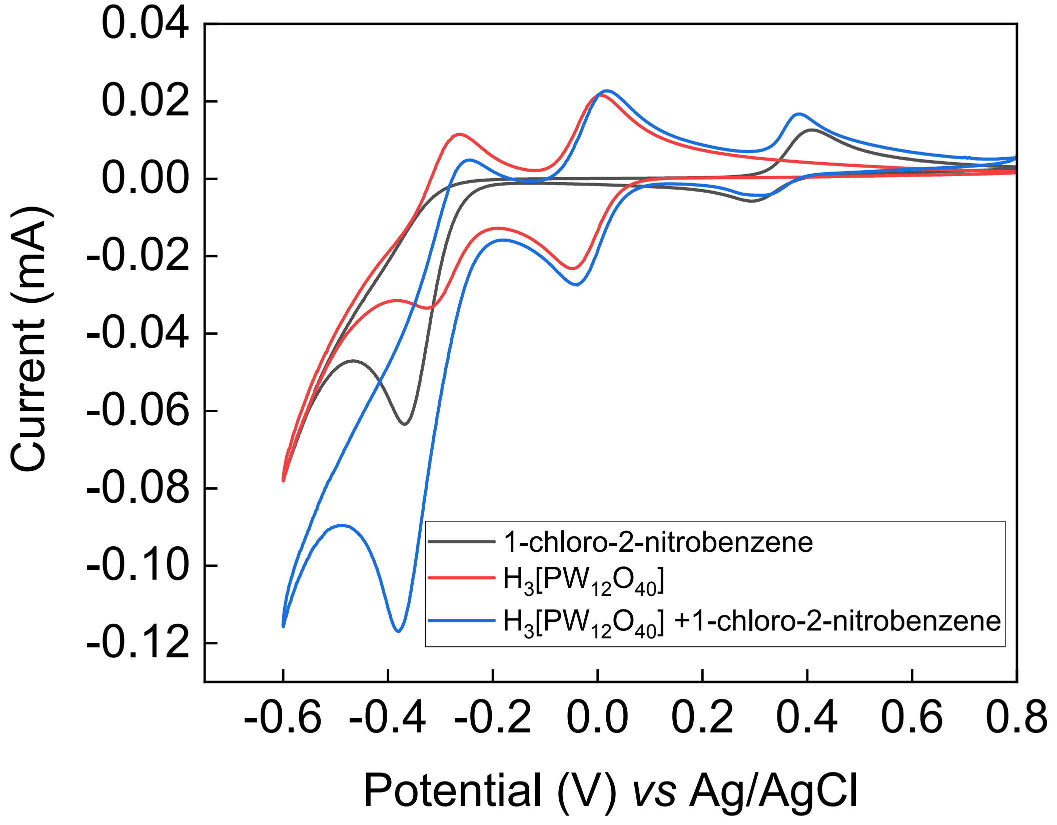

Figure S2: Cyclic voltammogram of the starting material 1-chloro-2-nitrobenzene in 1 M aqueous H_3PO_4, using 9.74×10^{-5} mol of both the nitroarene and the redox mediator. A glassy carbon working electrode (surface area = 0.071 cm^2), a Pt wire counter electrode and a Ag/AgCl reference electrode were used. Scan rate 10 mV/s.

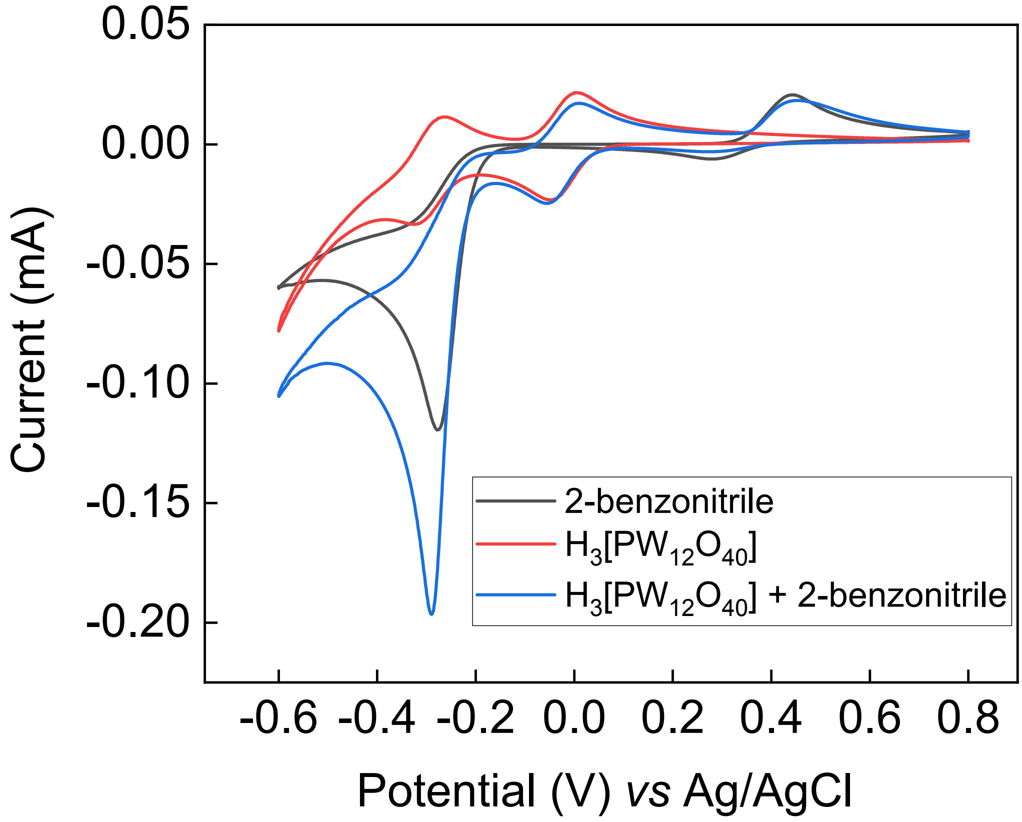

Figure S3: Cyclic voltammogram of the starting material 2-nitrobenzonitrile in 1 M aqueous H_3PO_4, using 9.74×10^{-5} mol of both the nitroarene and the redox mediator. A glassy carbon working electrode (surface area = 0.071 cm^2), a Pt wire counter electrode and a Ag/AgCl reference electrode were used. Scan rate 10 mV/s.

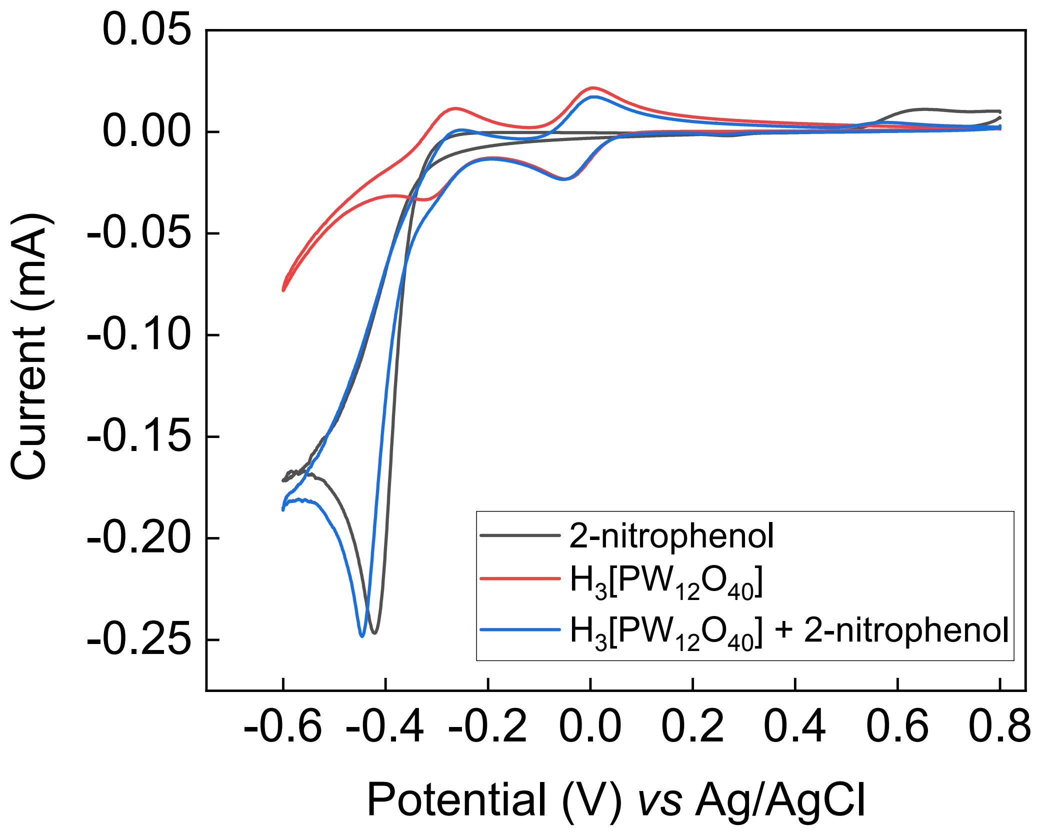

Figure S4: Cyclic voltammogram of the starting material 2-nitrophenol in 1 M aqueous H_3PO_4, using 9.74×10^{-5} mol of both the nitroarene and the redox mediator. A glassy carbon working electrode (surface area = 0.071 cm^2), a Pt wire counter electrode and a Ag/AgCl reference electrode were used. Scan rate 10 mV/s.

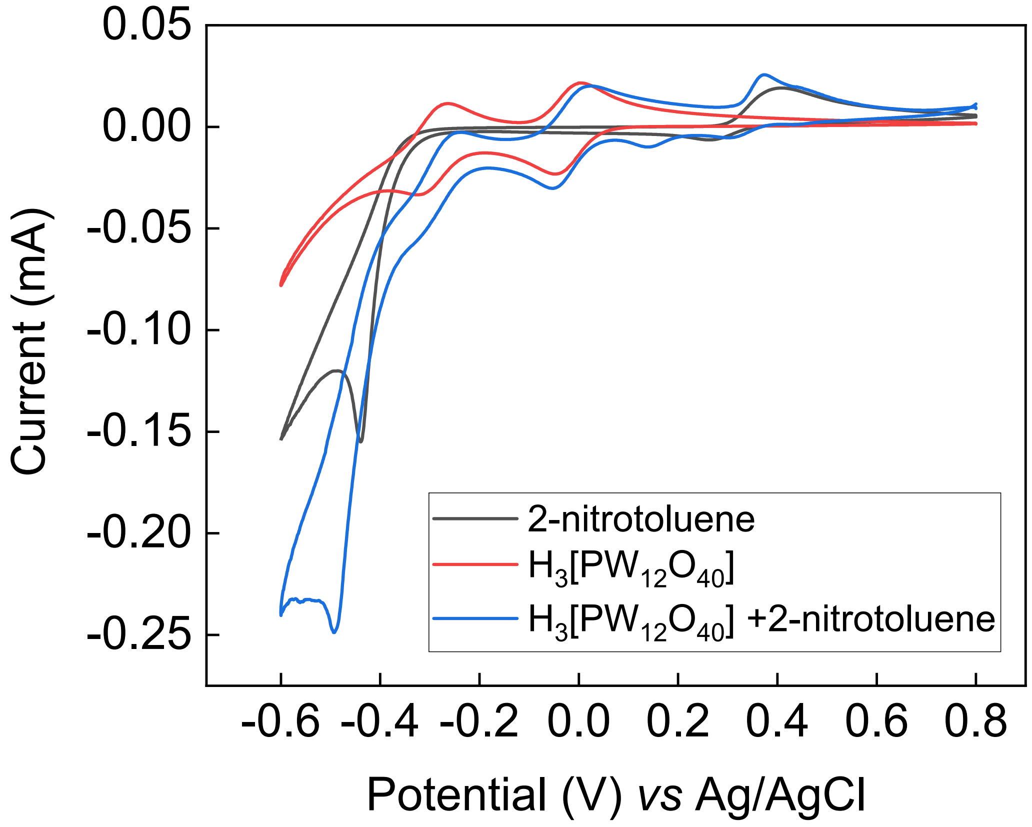

Figure S5: Cyclic voltammogram of the starting material 2-nitrotoluene in 1 M aqueous H_3PO_4, using 9.74×10^{-5} mol of both the nitroarene and the redox mediator. A glassy carbon working electrode (surface area = 0.071 cm^2), a Pt wire counter electrode and a Ag/AgCl reference electrode were used. Scan rate 10 mV/s.

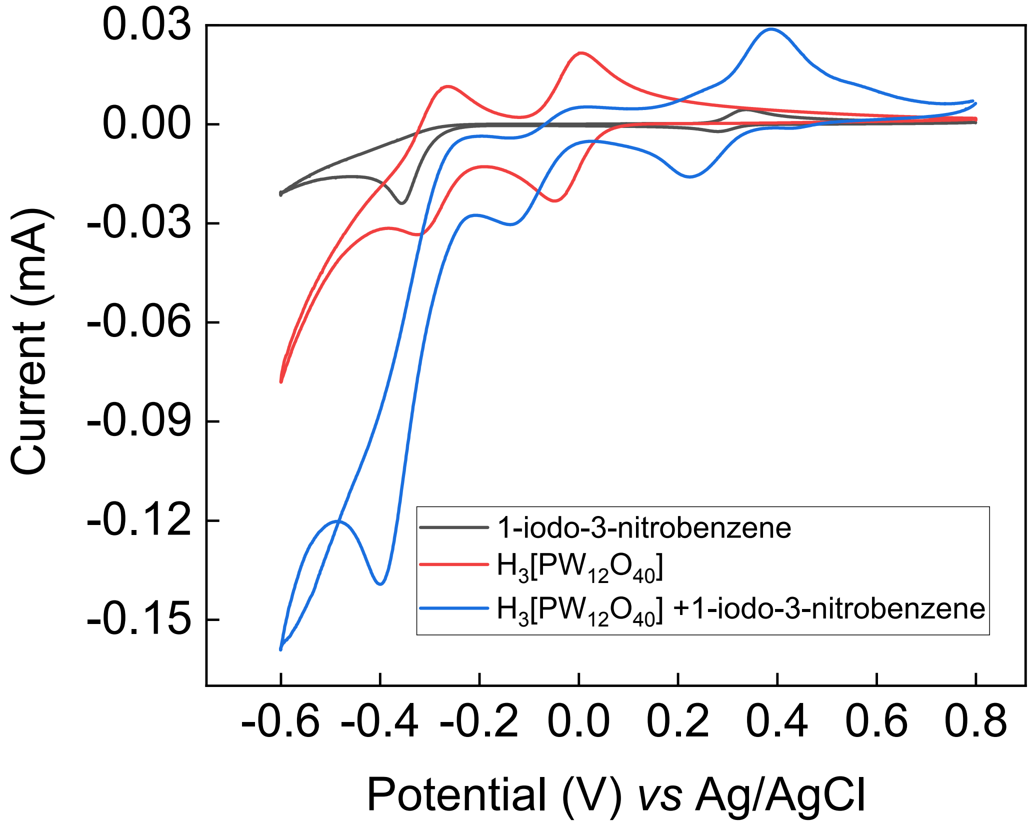

Figure S6: Cyclic voltammogram of the starting material 1-iodo-3-nitrobenzene in 1 M aqueous H_3PO_4, using 1.95×10^{-4} mol of starting material and 9.74×10^{-5} mol of the redox mediator. A glassy carbon working electrode (surface area = 0.071 cm^2), a Pt wire counter electrode and a Ag/AgCl reference electrode were used. Scan rate 10 mV/s.

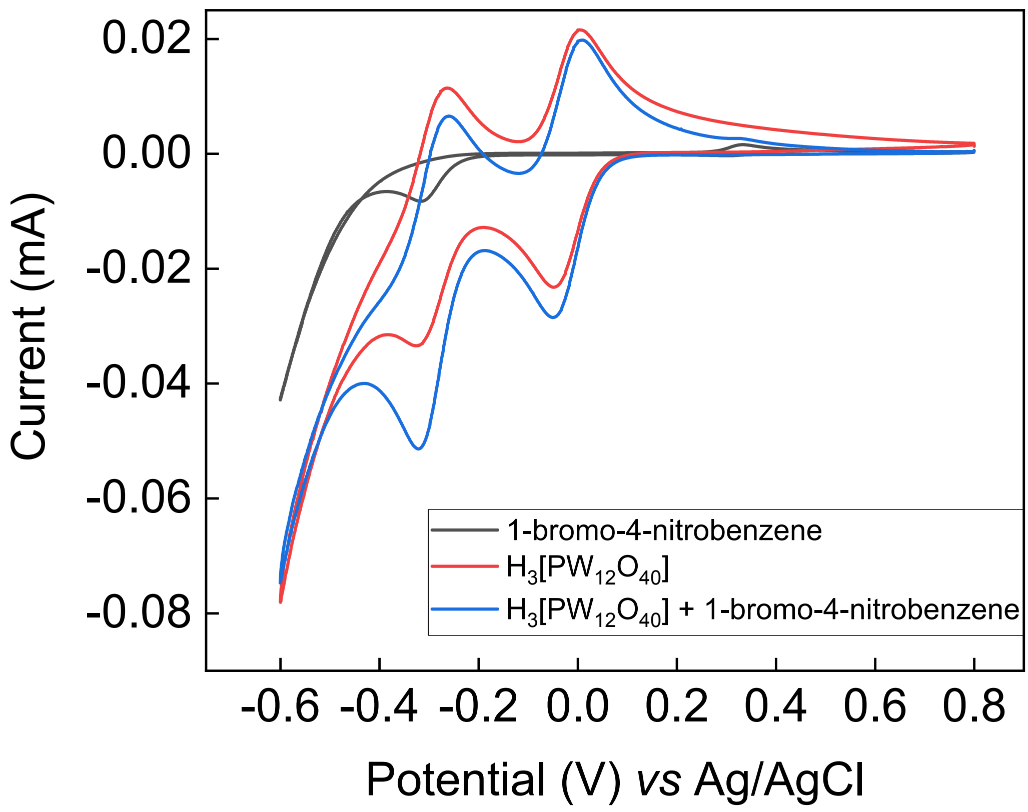

Figure S7: Cyclic voltammogram of the starting material 1-bromo-4-nitrobenzene in 1 M aqueous H_3PO_4, using 1.95×10^{-4} mol of starting material and 9.74×10^{-5} mol of the redox mediator. A glassy carbon working electrode (surface area = 0.071 cm^2), a Pt wire counter electrode and a Ag/AgCl reference electrode were used. Scan rate 10 mV/s.

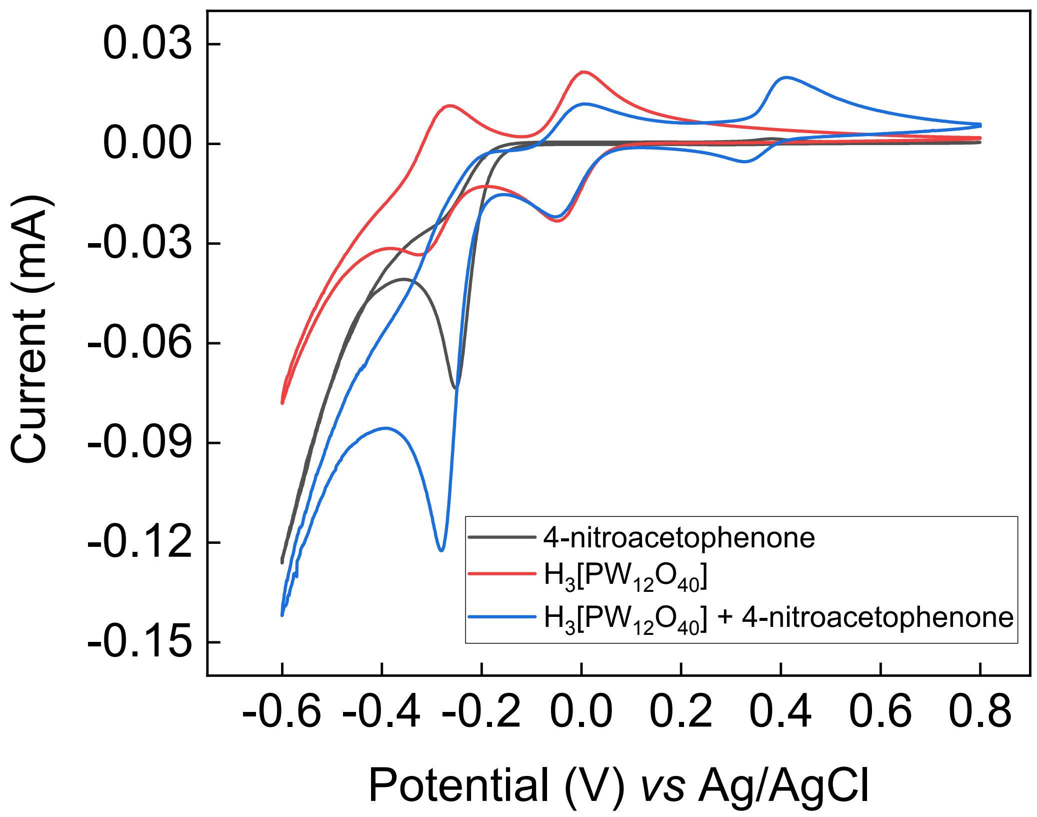

Figure S8: Cyclic voltammogram of the starting material 4-nitroacetophenone in 1 M aqueous H_3PO_4, using 9.74×10^{-5} mol of both the nitroarene and the redox mediator. A glassy carbon working electrode (surface area = 0.071 cm^2), a Pt wire counter electrode and a Ag/AgCl reference electrode were used. Scan rate 10 mV/s.

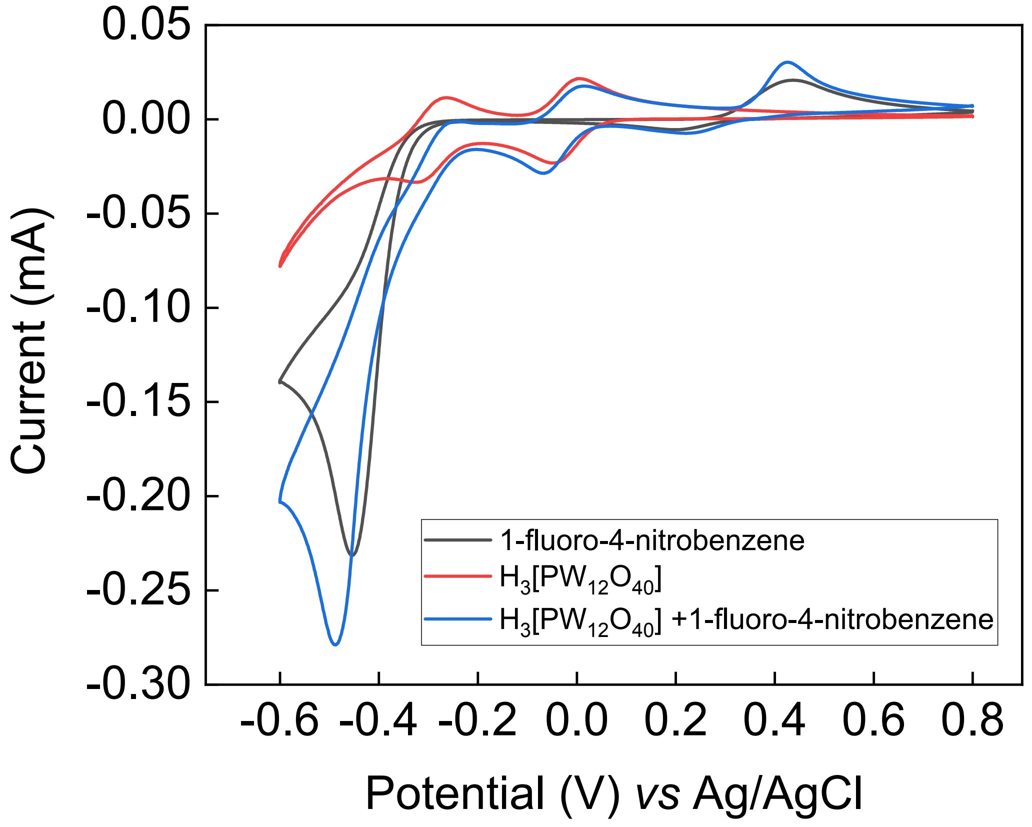

Figure S9: Cyclic voltammogram of the starting material 1-fluoro-4-nitrobenzene in 1 M aqueous H_3PO_4, using 1.95×10^{-4} mol of starting material and 9.74×10^{-5} mol of the redox mediator. A glassy carbon working electrode (surface area = 0.071 cm^2), a Pt wire counter electrode and a Ag/AgCl reference electrode were used. Scan rate 10 mV/s.

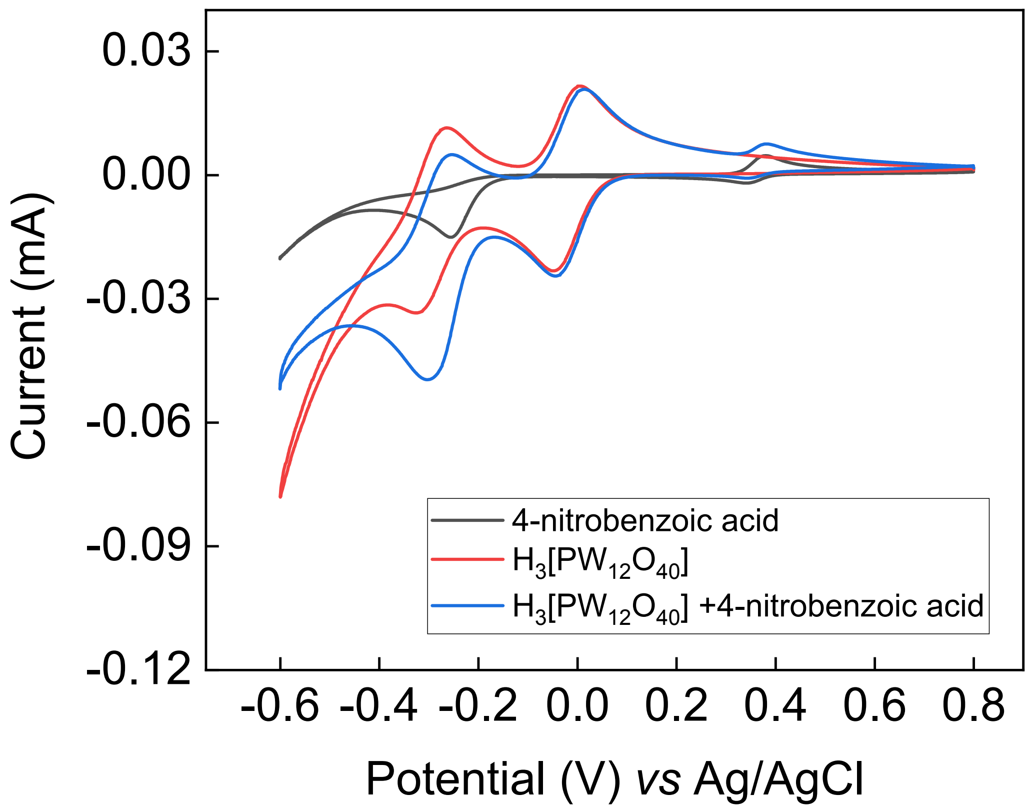

Figure S10: Cyclic voltammogram of the starting material 4-nitrobenzoic acid in 1 M aqueous H_3PO_4, using 1.95×10^{-4} mol of starting material and 9.74×10^{-5} mol of the redox mediator. A glassy carbon working electrode (surface area = 0.071 cm^2), a Pt wire counter electrode and a Ag/AgCl reference electrode were used. Scan rate 10 mV/s.

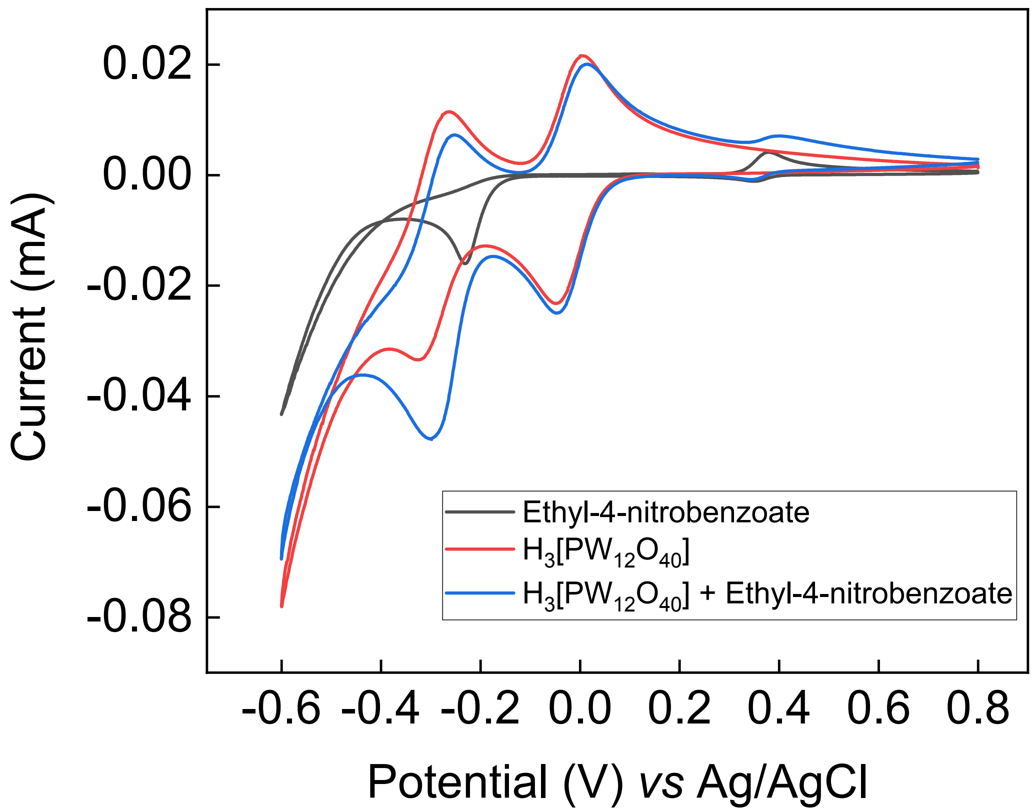

Figure S11: Cyclic voltammogram of the starting material ethyl-4-nitrobenzoate in 1 M aqueous H_3PO_4, using 9.74×10^{-5} mol of both the nitroarene and the redox mediator. A glassy carbon working electrode (surface area = 0.071 cm^2), a Pt wire counter electrode and a Ag/AgCl reference electrode were used. Scan rate 10 mV/s.

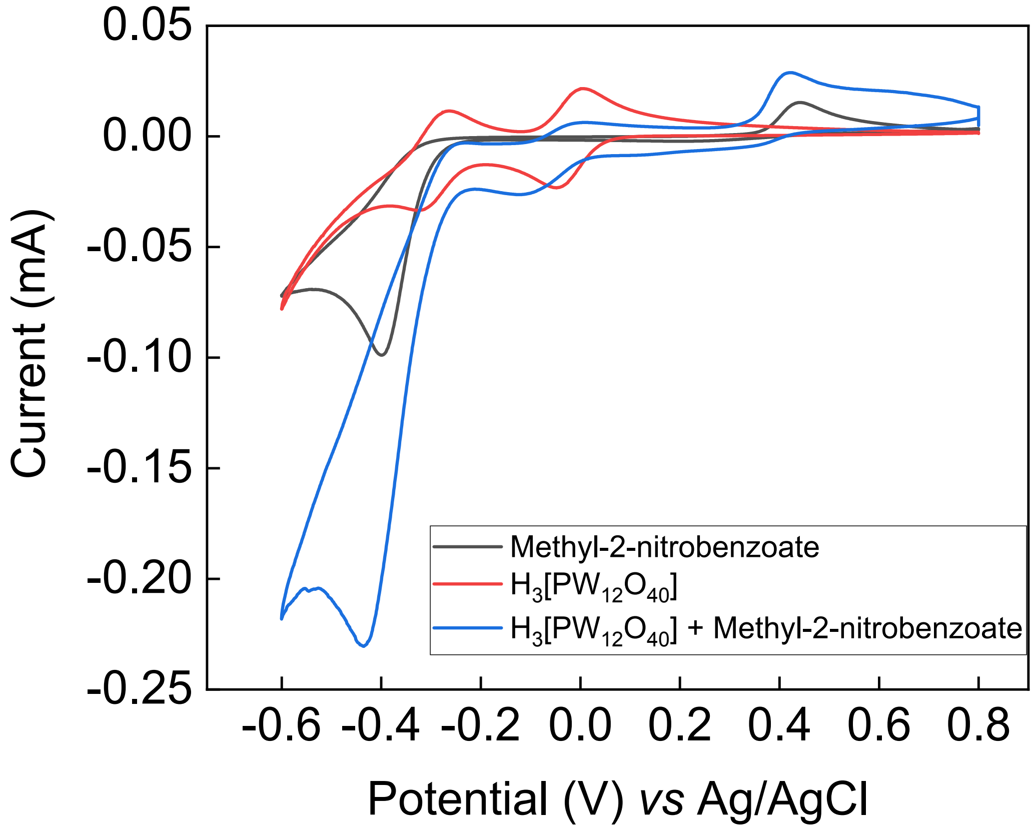

Figure S12: Cyclic voltammogram of the starting material methyl-2-nitrobenzoate in 1 M aqueous H_3PO_4, using 1.95 × 10^{-4} mol of starting material and 9.74 × 10^{-5} mol the redox mediator. A glassy carbon working electrode (surface area = 0.071 cm^2), a Pt wire counter electrode and a Ag/AgCl reference electrode were used. Scan rate 10 mV/s.

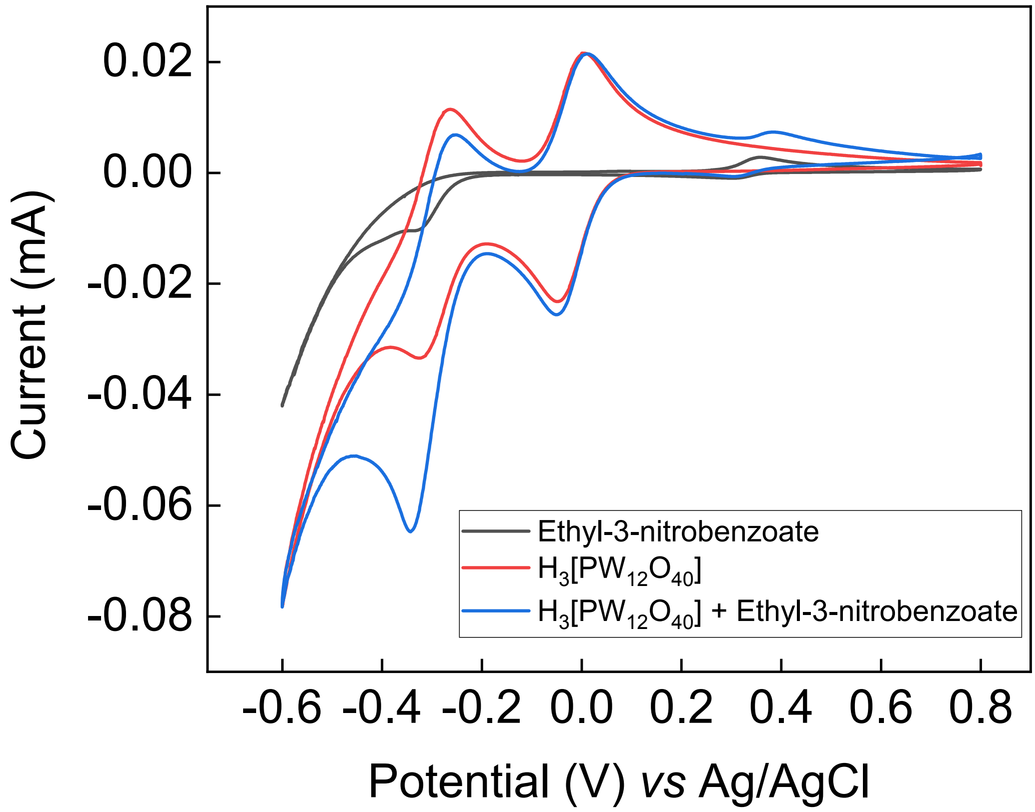

Figure S13: Cyclic voltammogram of the starting material ethyl-3-nitrobenzoate in 1 M aqueous H_3PO_4, using 9.74×10^{-5} mol of both the nitroarene and the redox mediator. A glassy carbon working electrode (surface area = 0.071 cm^2), a Pt wire counter electrode and a Ag/AgCl reference electrode were used. Scan rate 10 mV/s.

NMR data

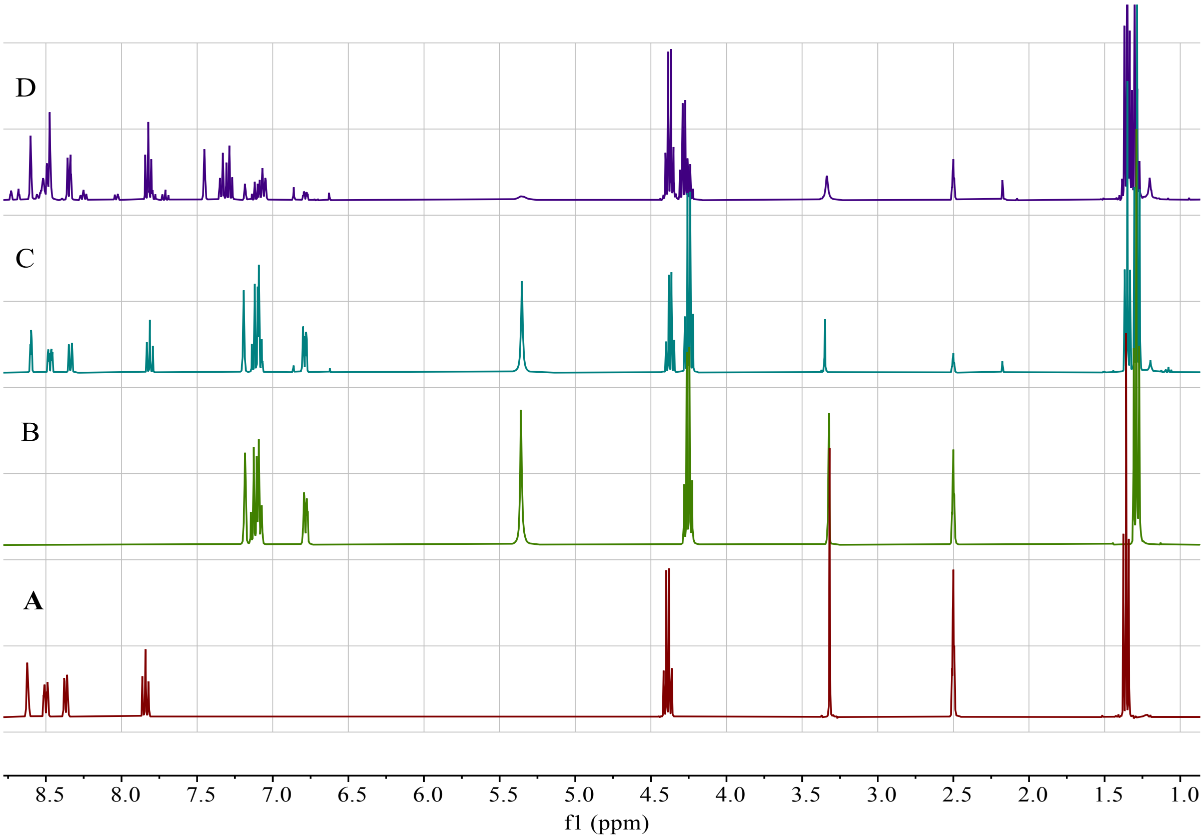

Figure S14: Reduction of ethyl-3-nitrobenzoate. ^{1}H NMR spectra of the ethyl-3-nitrobenzoate starting material (A), a sample of pure ethyl-3-aminobenzoate (B), the spectrum of the electrocatalytic reaction medium after extraction and concentration (C) and the spectrum of the extracted and concentrated reaction medium from a direct (i.e. non-mediated) electrochemical reduction of ethyl-3-nitrobenzoate (D). All spectra were obtained in DMSO-d_6.

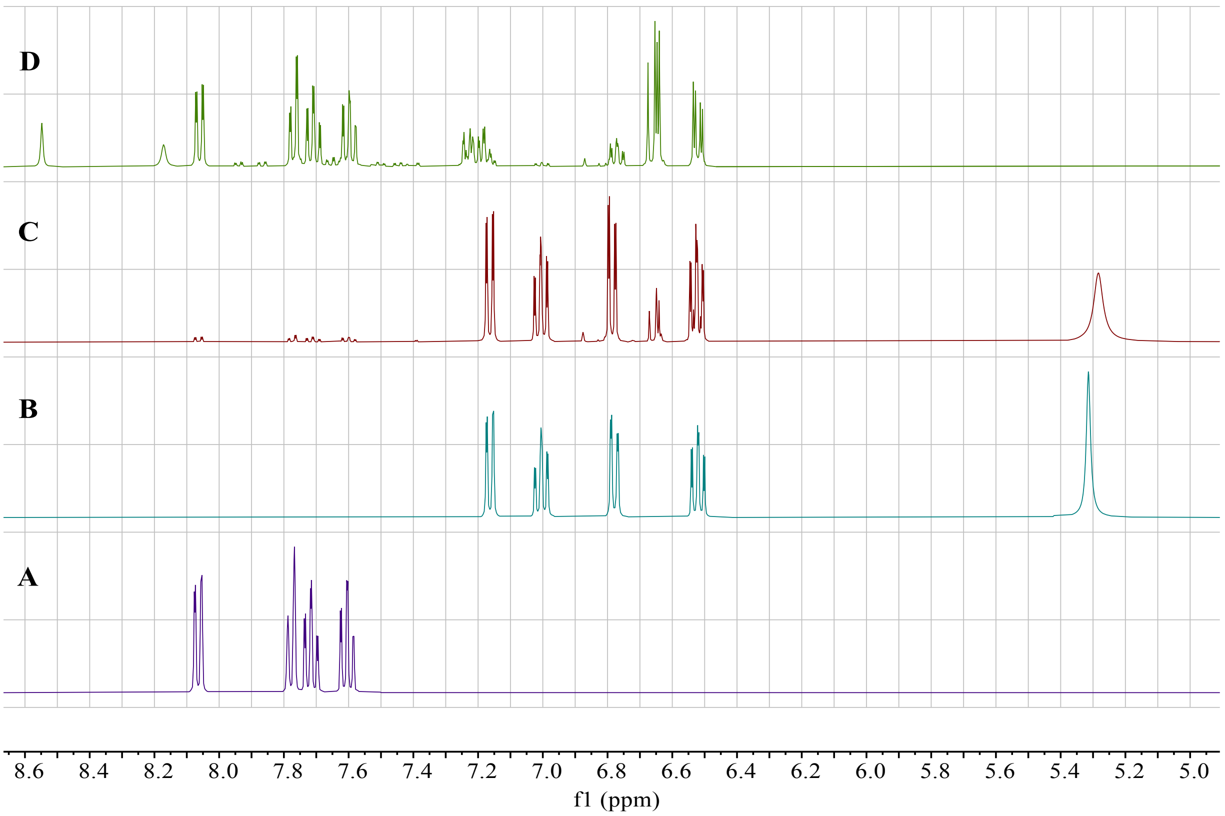

Figure S15: Reduction of 1-chloro-2-nitrobenzene. ^{1}H NMR spectra of the 1-chloro-2-nitrobenzene starting material (A), a sample of pure 2-chloroaniline (B), the spectrum of the electrocatalytic reaction medium after extraction and concentration (C) and the spectrum of the extracted and concentrated reaction medium from a direct (i.e. non-mediated) electrochemical reduction of 1-chloro-2-nitrobenzene (D). All spectra were obtained in DMSO-d_6.

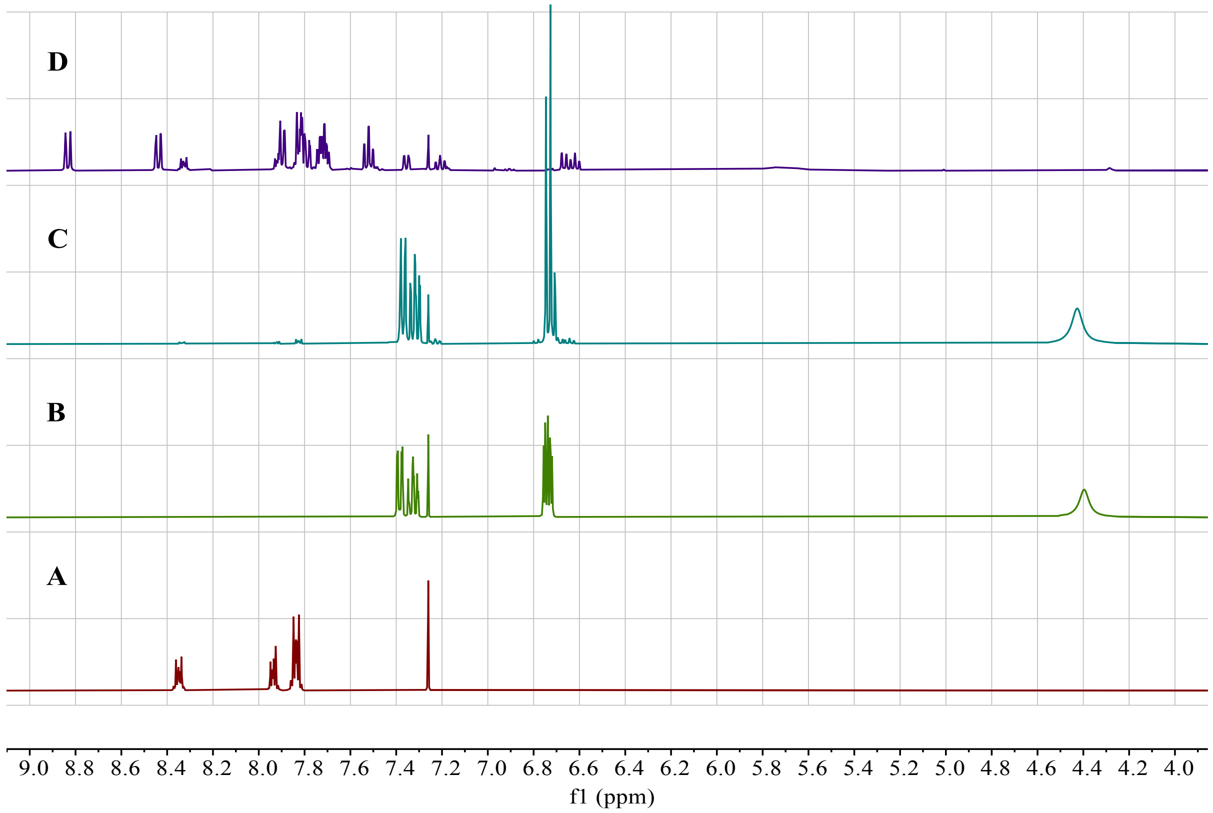

Figure S16: Reduction of 2-nitrobenzonitrile. ^{1}H NMR spectra of the 2-nitrobenzonitrile starting material (A), a sample of 2-aminobenzonitrile (B), the spectrum of the electrocatalytic reaction medium after extraction and concentration (C) and the spectrum of the extracted and concentrated reaction medium from a direct (i.e. non-mediated) electrochemical reduction of 2-nitrobenzonitrile (D). All spectra were obtained in $CDCl_3$.

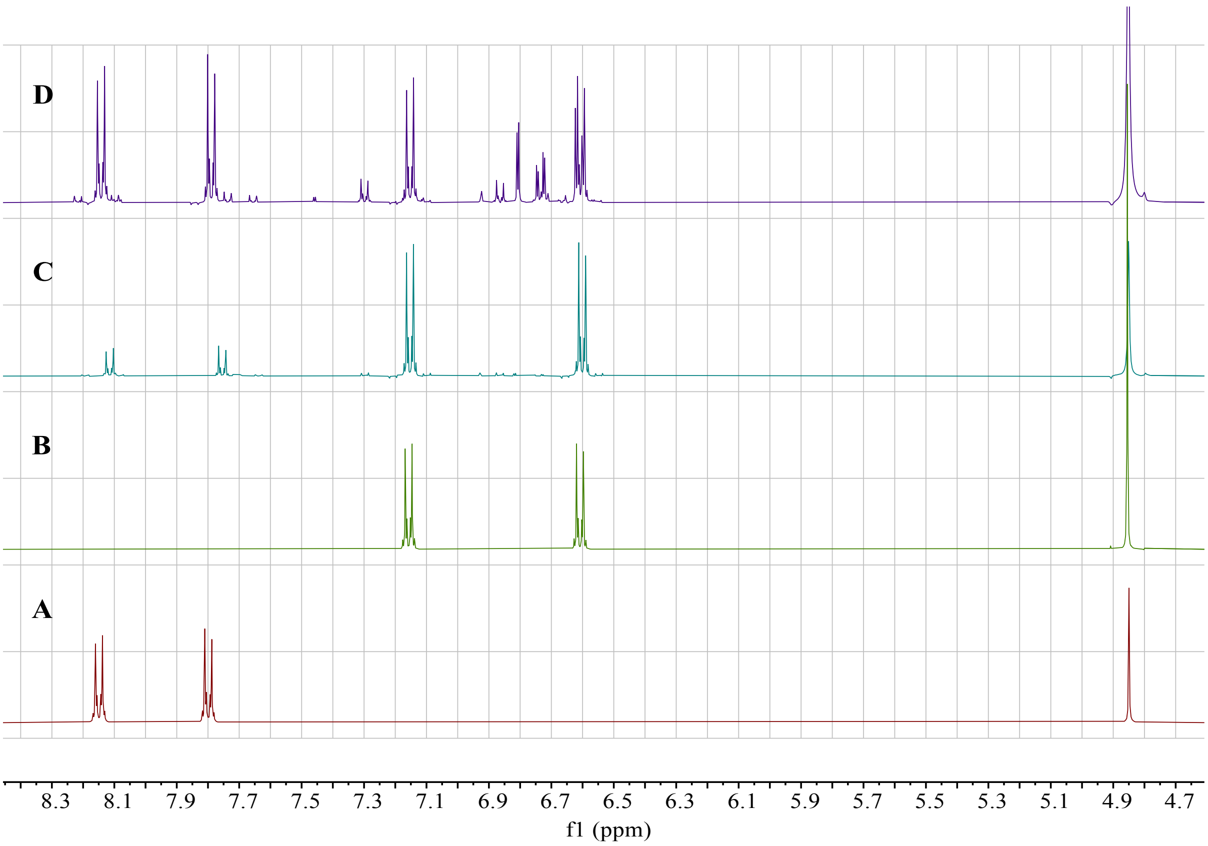

Figure S17: Reduction of 1-bromo-4-nitrobenzene. ^{1}H NMR spectra of the 1-bromo-4-nitrobenzene starting material (A), a sample of 4-bromoaniline (B), the spectrum of the electrocatalytic reaction medium after extraction and concentration (C) and the spectrum of the extracted and concentrated reaction medium from a direct (i.e. non-mediated) electrochemical reduction of 1-bromo-4-nitrobenzene (D). All spectra were obtained in MeOD.

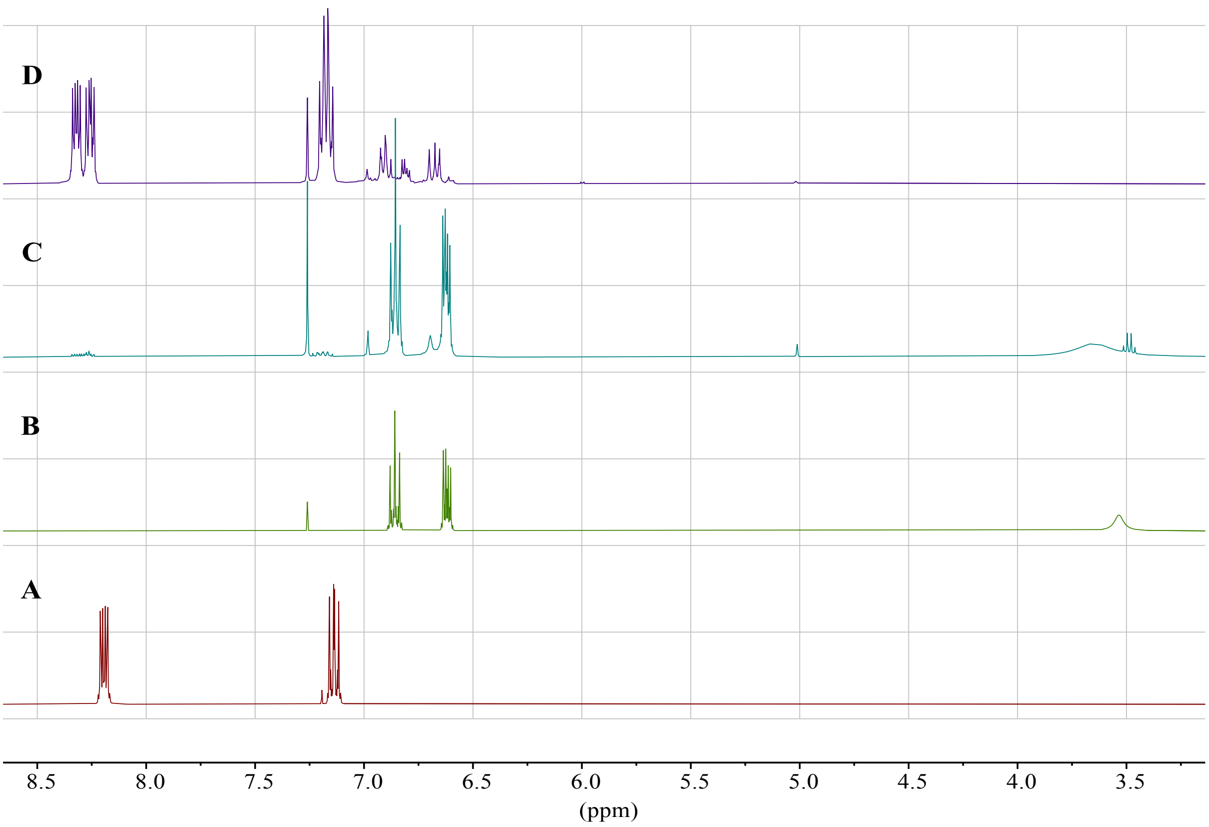

Figure S18: Reduction of 1-fluoro-4-nitrobenzene. ^{1}H NMR spectra of the 1-fluoro-4-nitrobenzene starting material (A), a sample of 4-fluoroaniline (B), the spectrum of the electrocatalytic reaction medium after extraction and concentration (C) and the spectrum of the extracted and concentrated reaction medium from a direct (i.e. non-mediated) electrochemical reduction of 1-fluoro-4-nitrobenzene (D). All spectra were obtained in $CDCl_3$.

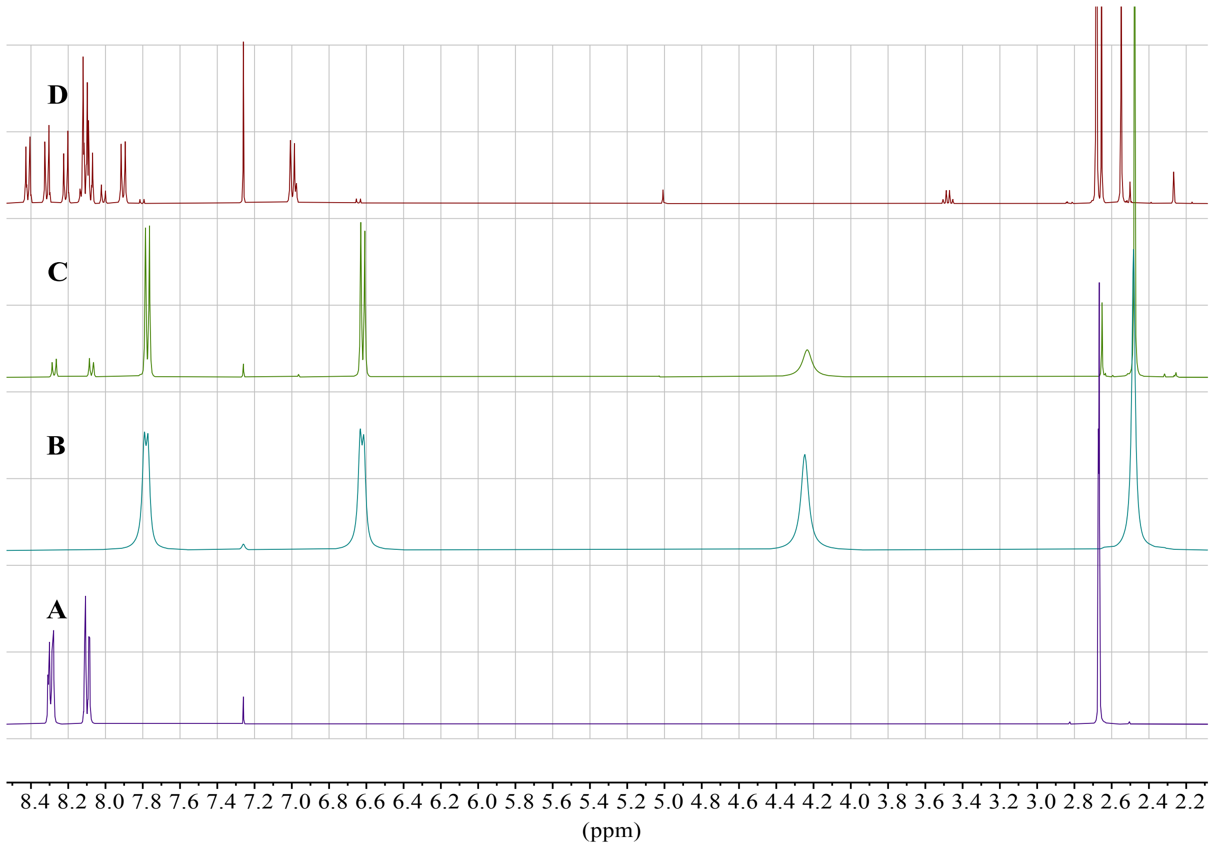

Figure S19: Reduction of 4-nitroacetophenone. ^{1}H NMR spectra of the 4-nitroacetophenone starting material (A), a sample of 4-aminoacetophenone (B), the spectrum of the electrocatalytic reaction medium after extraction and concentration (C) and the spectrum of the extracted and concentrated reaction medium from a direct (i.e. non-mediated) electrochemical reduction of 4-nitroacetophenone (D). All spectra were obtained in $CDCl_3$.

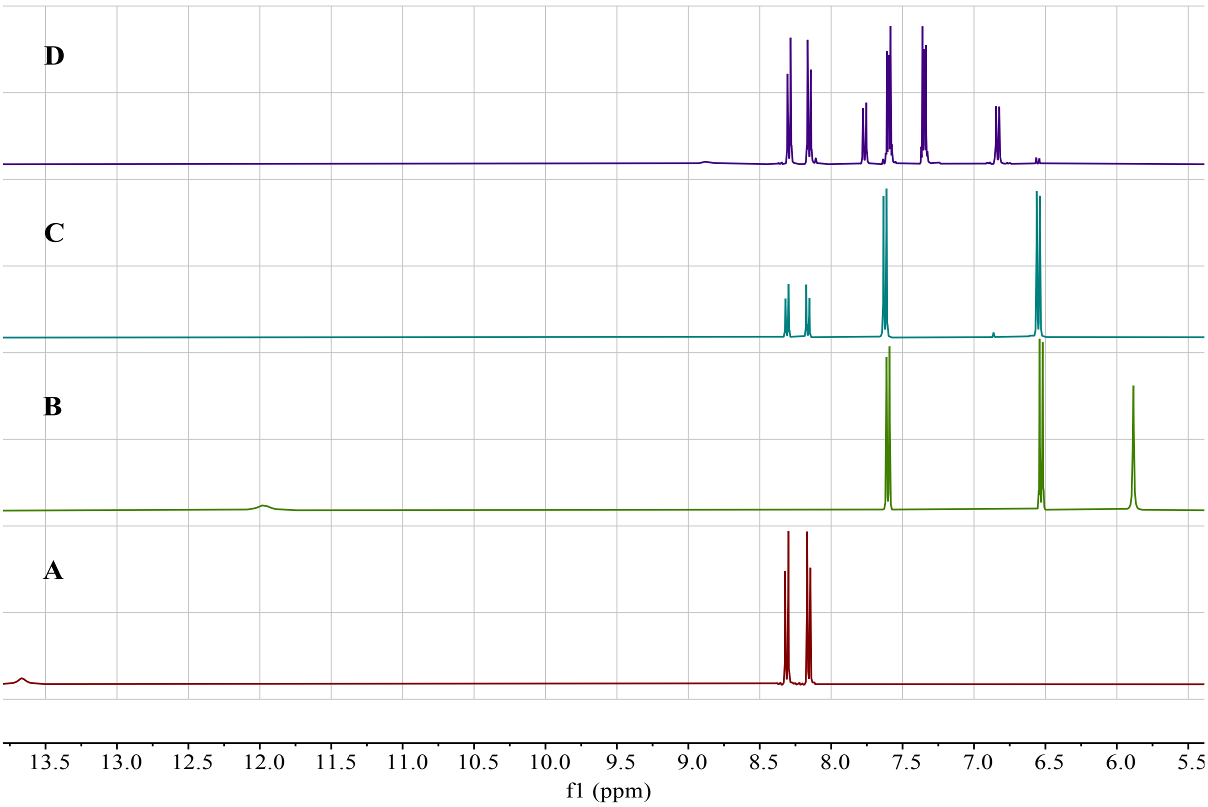

Figure S20: Reduction of 4-nitrobenzoic acid. ^{1}H NMR spectra of the 4-nitrobenzoic acid starting material (A), a sample of 4-aminobenzoic acid (B), the spectrum of the electrocatalytic reaction medium after extraction and concentration (C) and the spectrum of the extracted and concentrated reaction medium from a direct (i.e. non-mediated) electrochemical reduction of 4-nitrobenzoic acid (D). All spectra were obtained in DMSO-d_6.

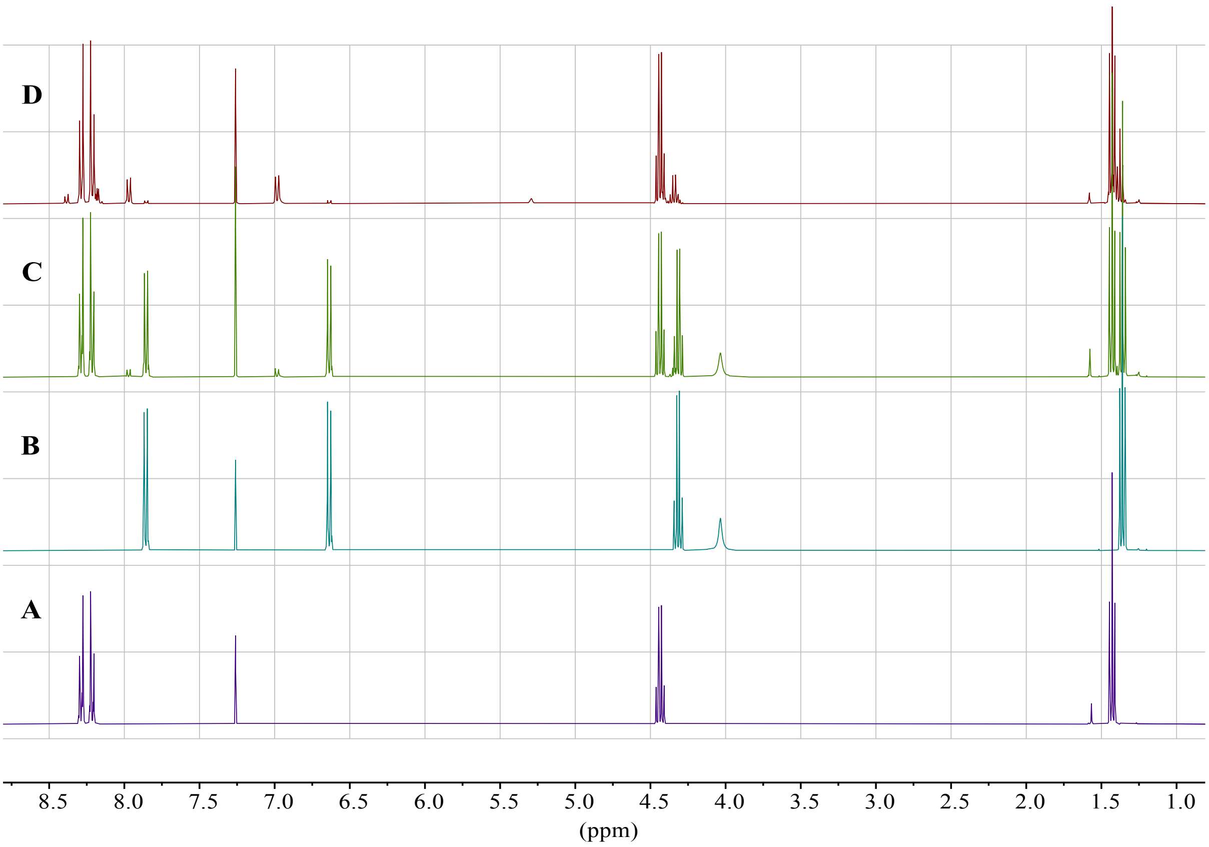

Figure S21: Reduction of ethyl-4-nitrobenzoate. ^{1}H NMR spectra of the ethyl-4-nitrobenzoate starting material (A), a sample of ethyl-4-aminobenzoate (B), the spectrum of the electrocatalytic reaction medium after extraction and concentration (C) and the spectrum of the extracted and concentrated reaction medium from a direct (i.e. non-mediated) electrochemical reduction of ethyl-4-nitrobenzoate (D). All spectra were obtained in $CDCl_3$.

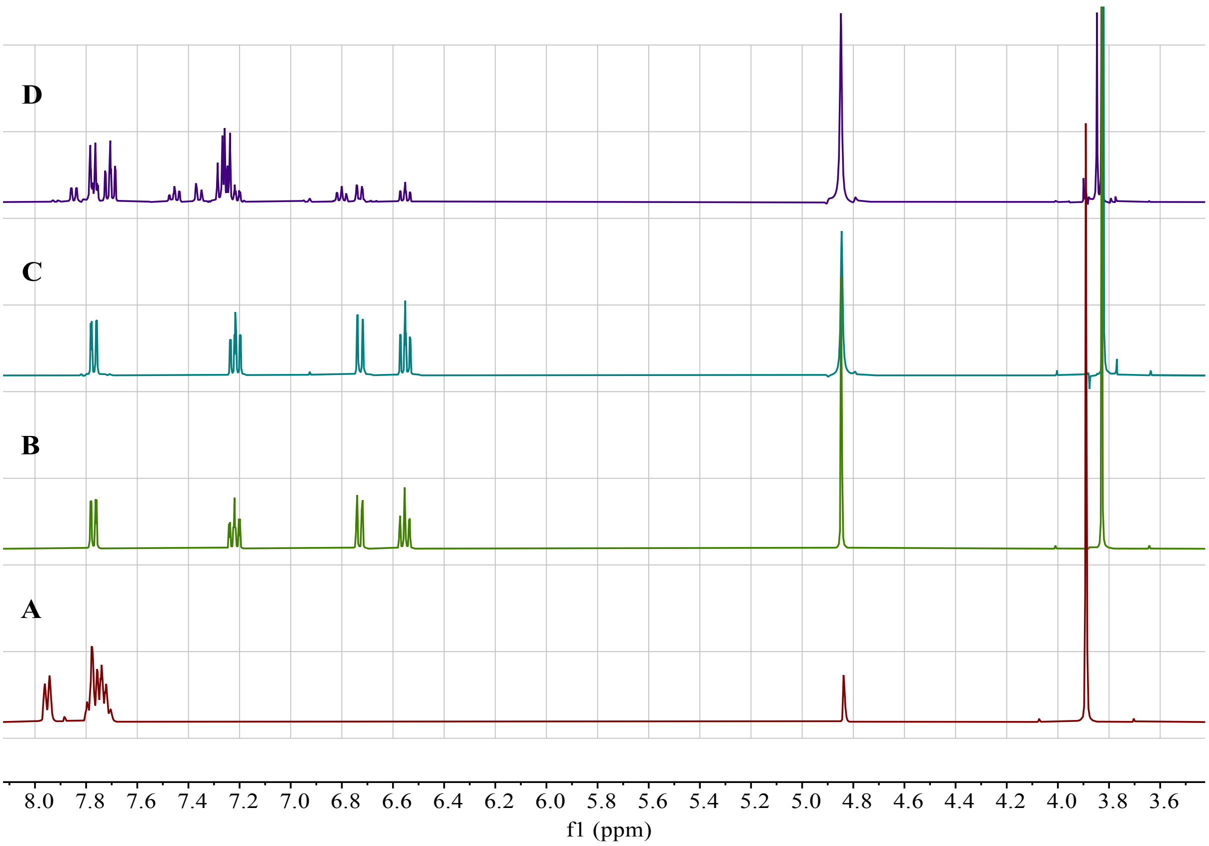

Figure S22: Reduction of methyl-2-nitrobenzoate. ^{1}H NMR spectra of the methyl-2-nitrobenzoate starting material (A), a sample of methyl-2-aminobenzoate (B), the spectrum of the electrocatalytic reaction medium after extraction and concentration (C) and the spectrum of the extracted and concentrated reaction medium from a direct (i.e. non-mediated) electrochemical reduction of methyl-2-nitrobenzoate (D). All spectra were obtained in MeOD.

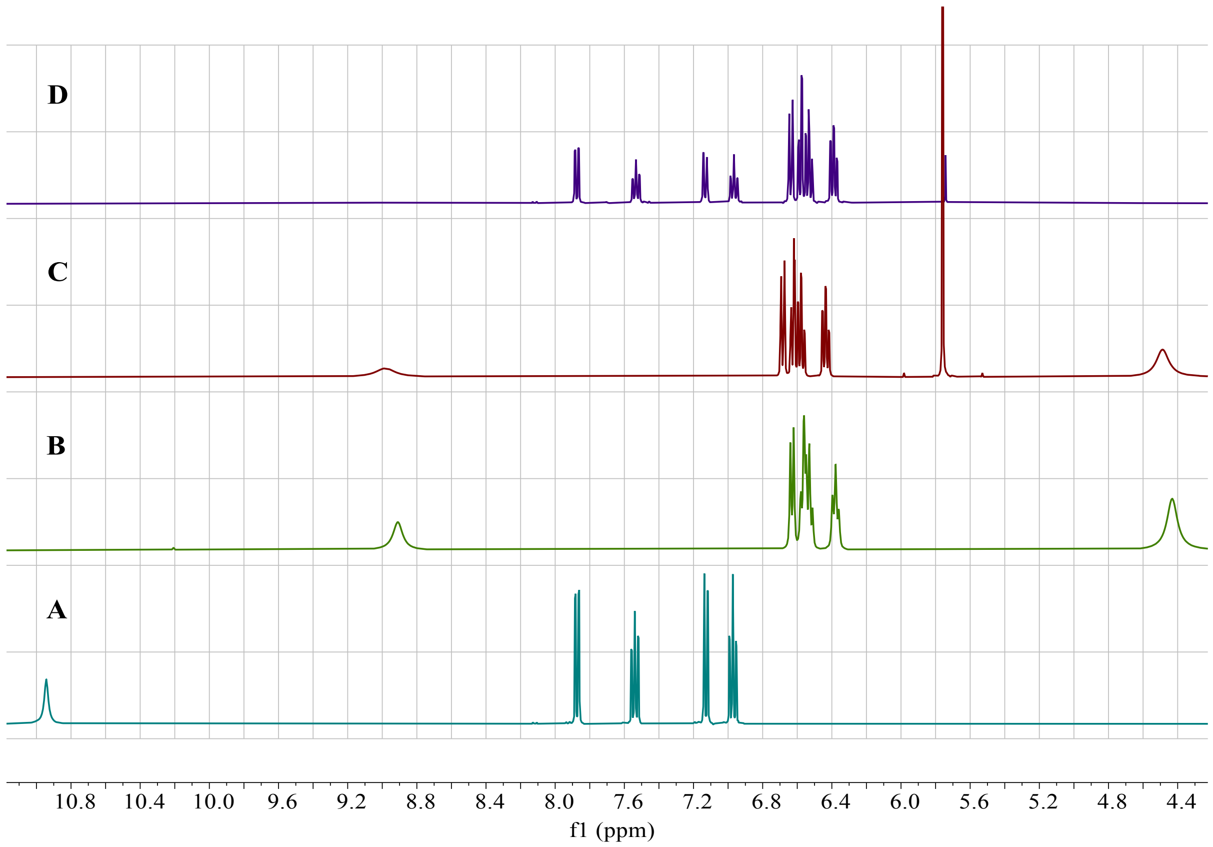

Figure S23: Reduction of 2-nitrophenol. ^{1}H NMR spectra of the 2-nitrophenol starting material (A), a sample of 2-aminophenol (B), the spectrum of the electrocatalytic reaction medium after extraction and concentration (C) and the spectrum of the extracted and concentrated reaction medium from a direct (i.e. non-mediated) electrochemical reduction of 2-nitrophenol (D). All spectra were obtained in DMSO-d_6.

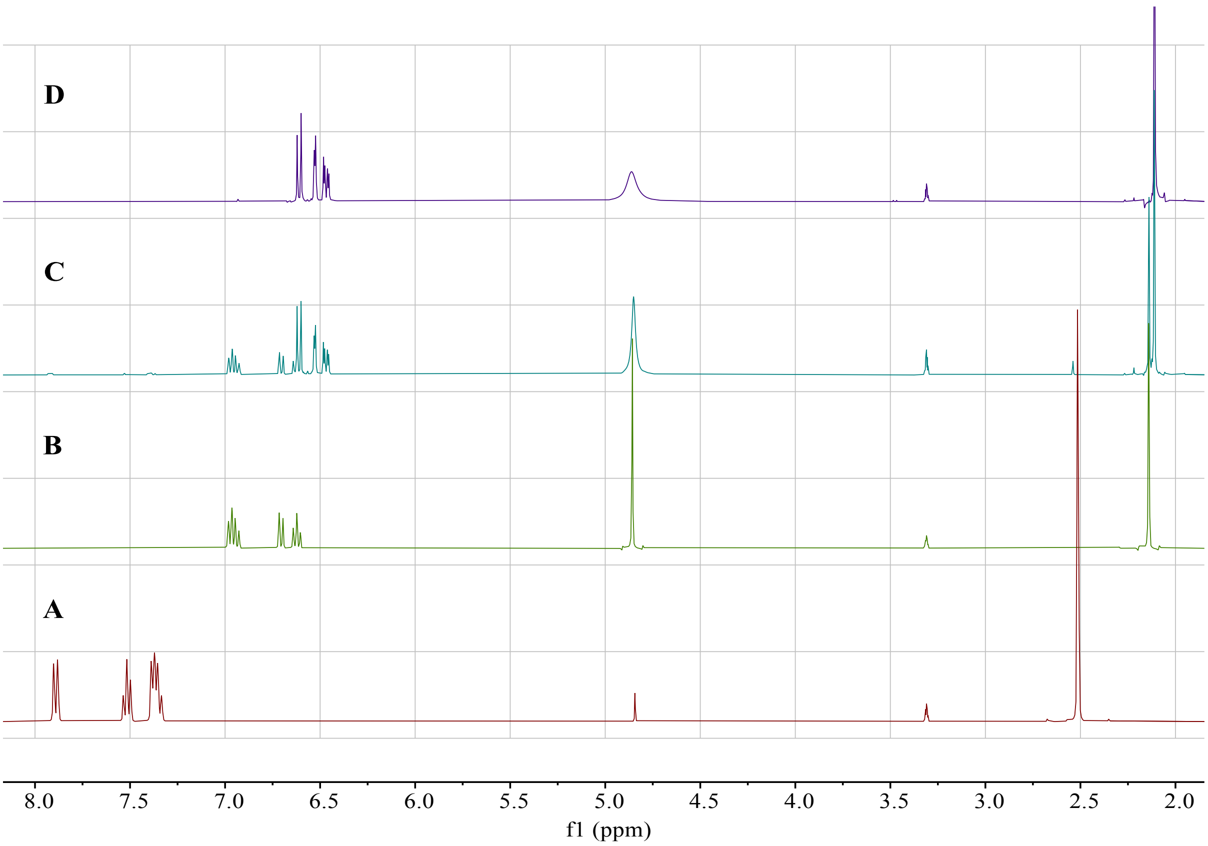

Figure S24: Reduction of 2-nitrotoluene. ^{1}H NMR spectra of the 2-nitrotoluene starting material (A), a sample of 2-aminotoluene (B), the spectrum of the electrocatalytic reaction medium after extraction and concentration (C) and the spectrum of the extracted and concentrated reaction medium from a direct (i.e. non-mediated) electrochemical reduction of 2-nitrotoluene (D). All spectra were obtained in MeOD.

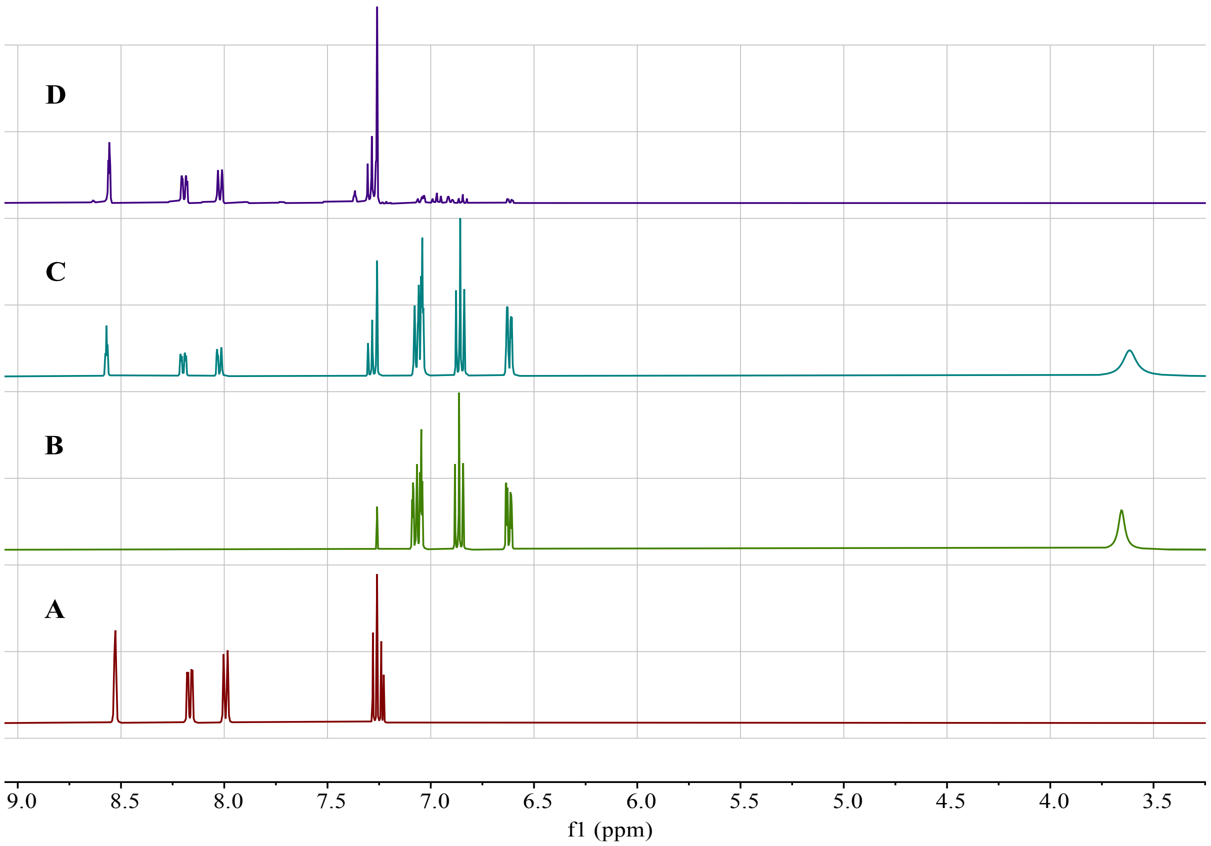

Figure S25: Reduction of 1-iodo-3-nitrobenzene. ^{1}H NMR spectra of the 1-iodo-3-nitrobenzene starting material (A), a sample of 3-iodoaniline (B), the spectrum of the electrocatalytic reaction medium after extraction and concentration (C) and the spectrum of the extracted and concentrated reaction medium from a direct (i.e. non-mediated) electrochemical reduction of 1-iodo-3-nitrobenzene (D). All spectra were obtained in MeOD.

Example Electrolysis Curve

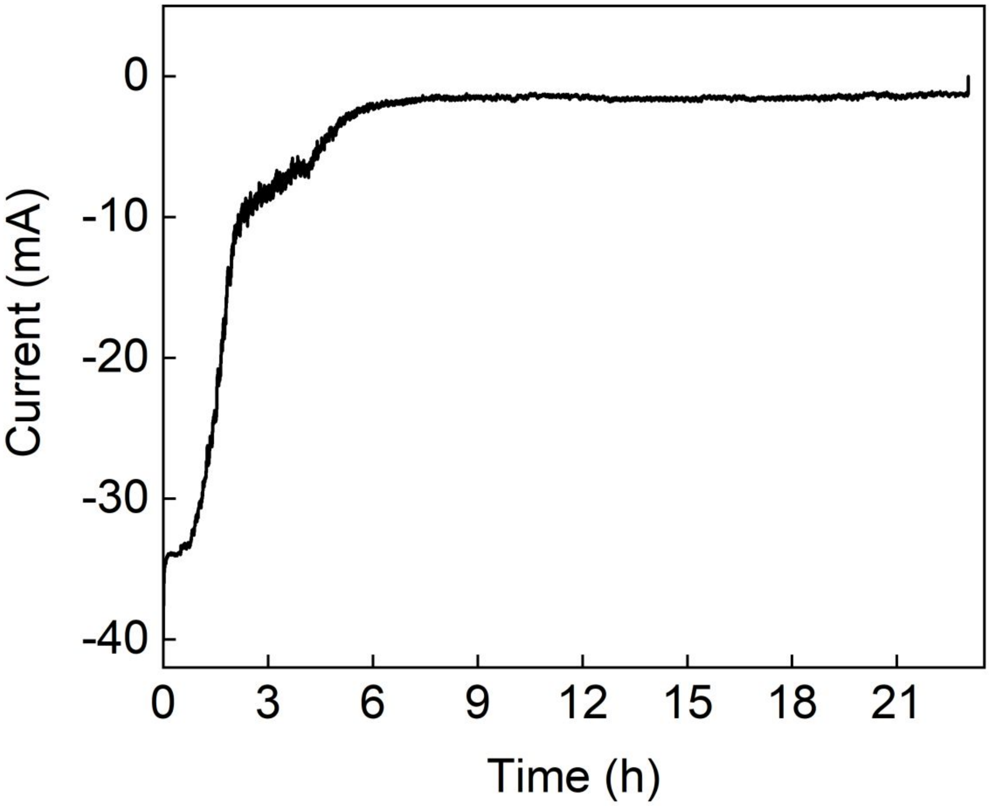

Figure S26: Example curve showing the bulk electrolysis of the nitroarene methyl-2-nitrobenzoate, where 0.088 g (4.87×10^{-4} moles) of the starting material were used together with 4.86×10^{-5} moles (10 mol%) of the polyoxometalate mediator in 30 mL electrolyte.

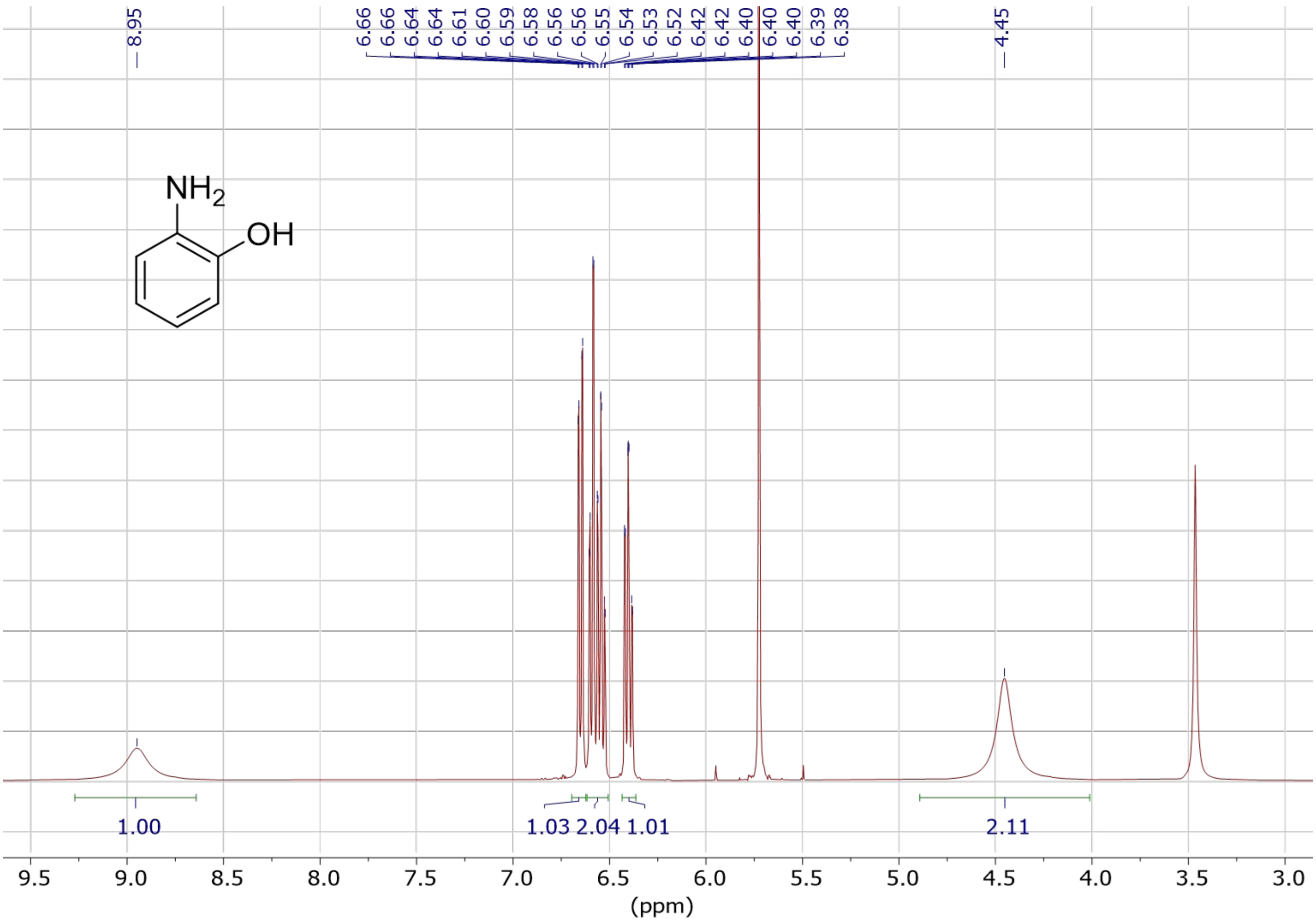

Figure S27: ^{1}H NMR (400 MHz, 298 K) spectrum of 2-aminophenol (Table 1, entry 1) from the electrocatalytic reaction medium after extraction and concentration. The spectrum was obtained in d_6-DMSO. The peak at 3.5 ppm is water and the peak at 5.6 ppm is dichloromethane (from the extraction).

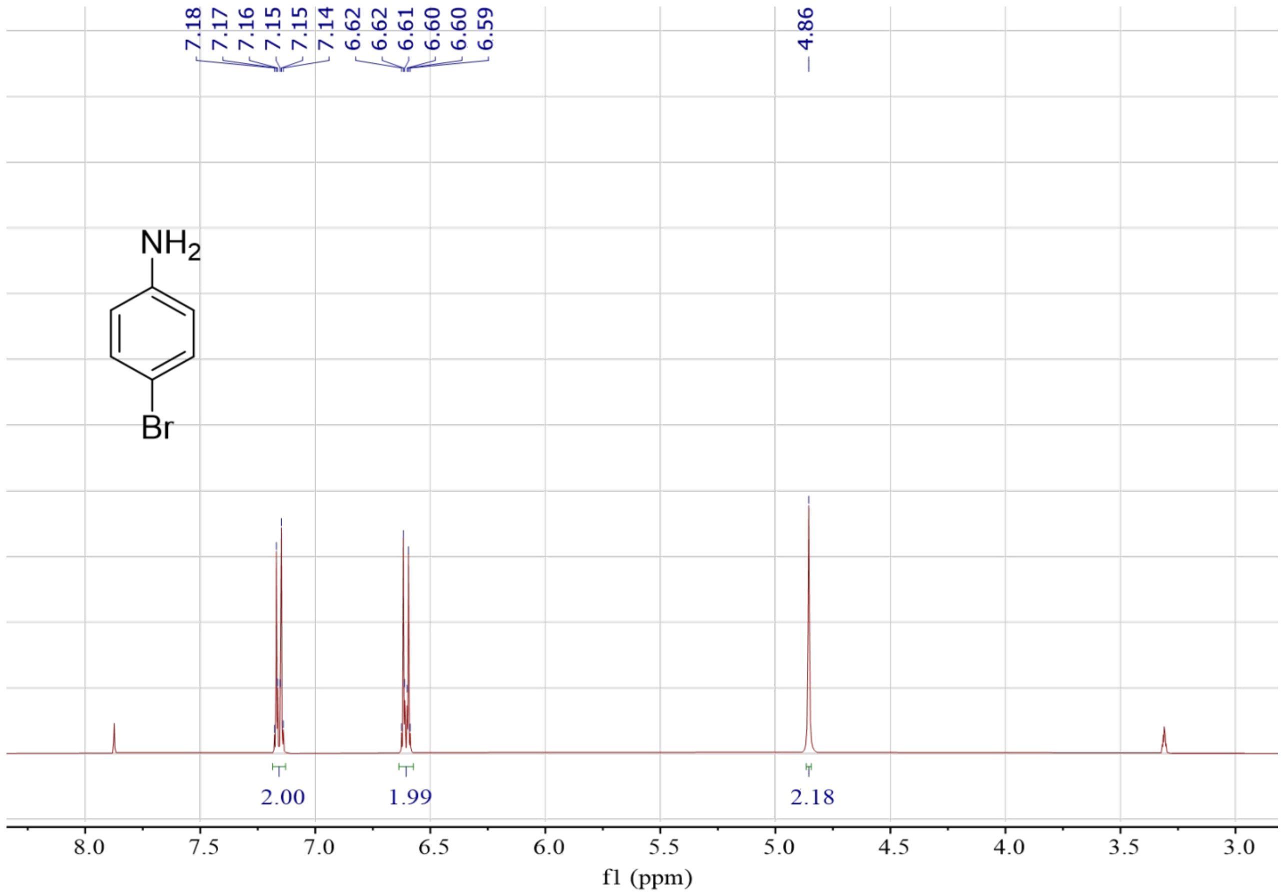

Figure S28: ^{1}H NMR (400 MHz, 298 K) spectrum of 4-bromo-aniline (Table 1, entry 2) from the electrocatalytic reaction medium after extraction, concentration, and purification. Spectrum was obtained in $CDCl_3$.

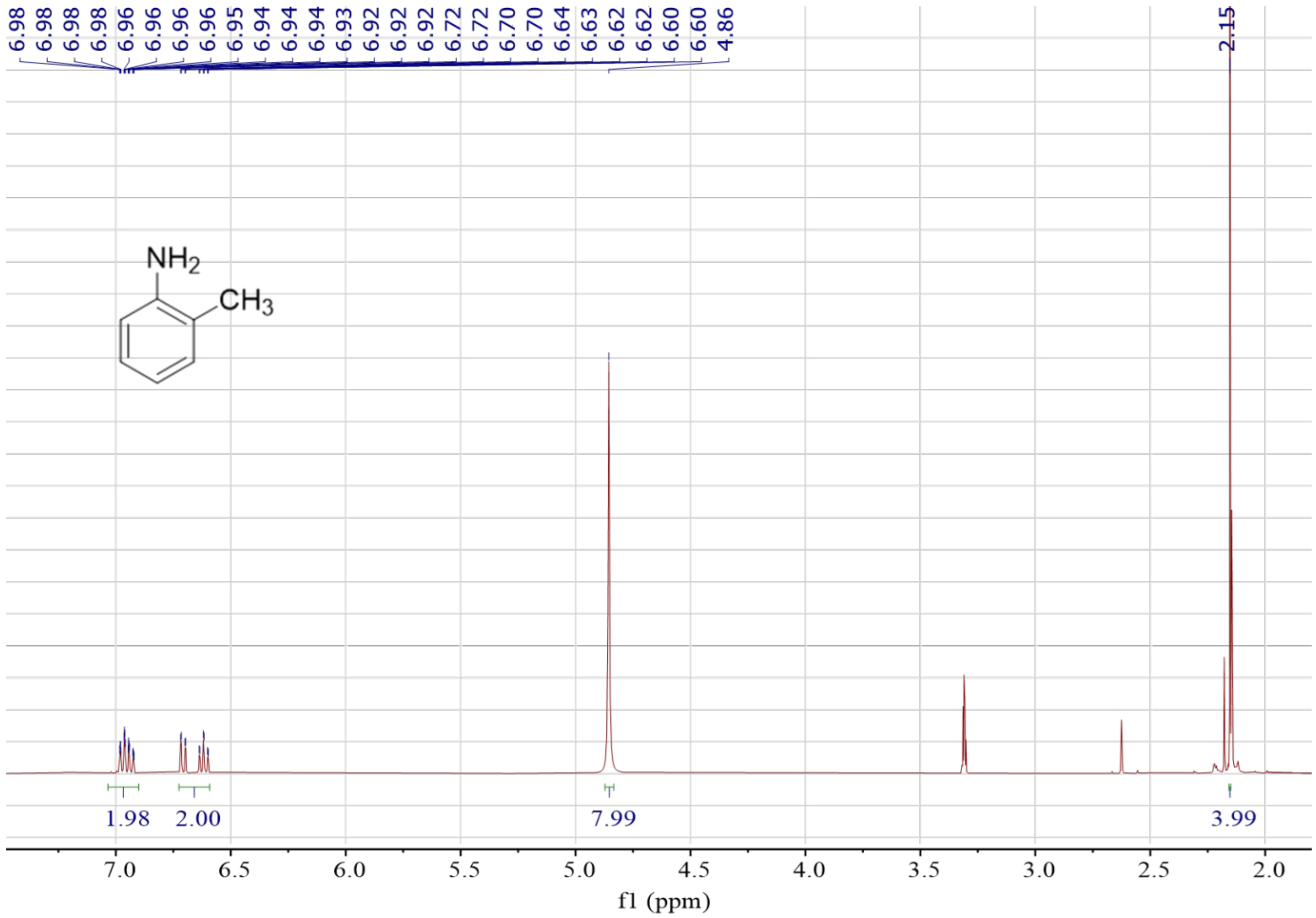

Figure S29: ^{1}H NMR (400 MHz, 298 K) spectrum of 2-methylaniline (Table 1, entry 3) from the electrocatalytic reaction medium after extraction, concentration, and purification. Spectrum was obtained in MeOD. The water peak at 4.8 ppm overlays the amine N-H peak.

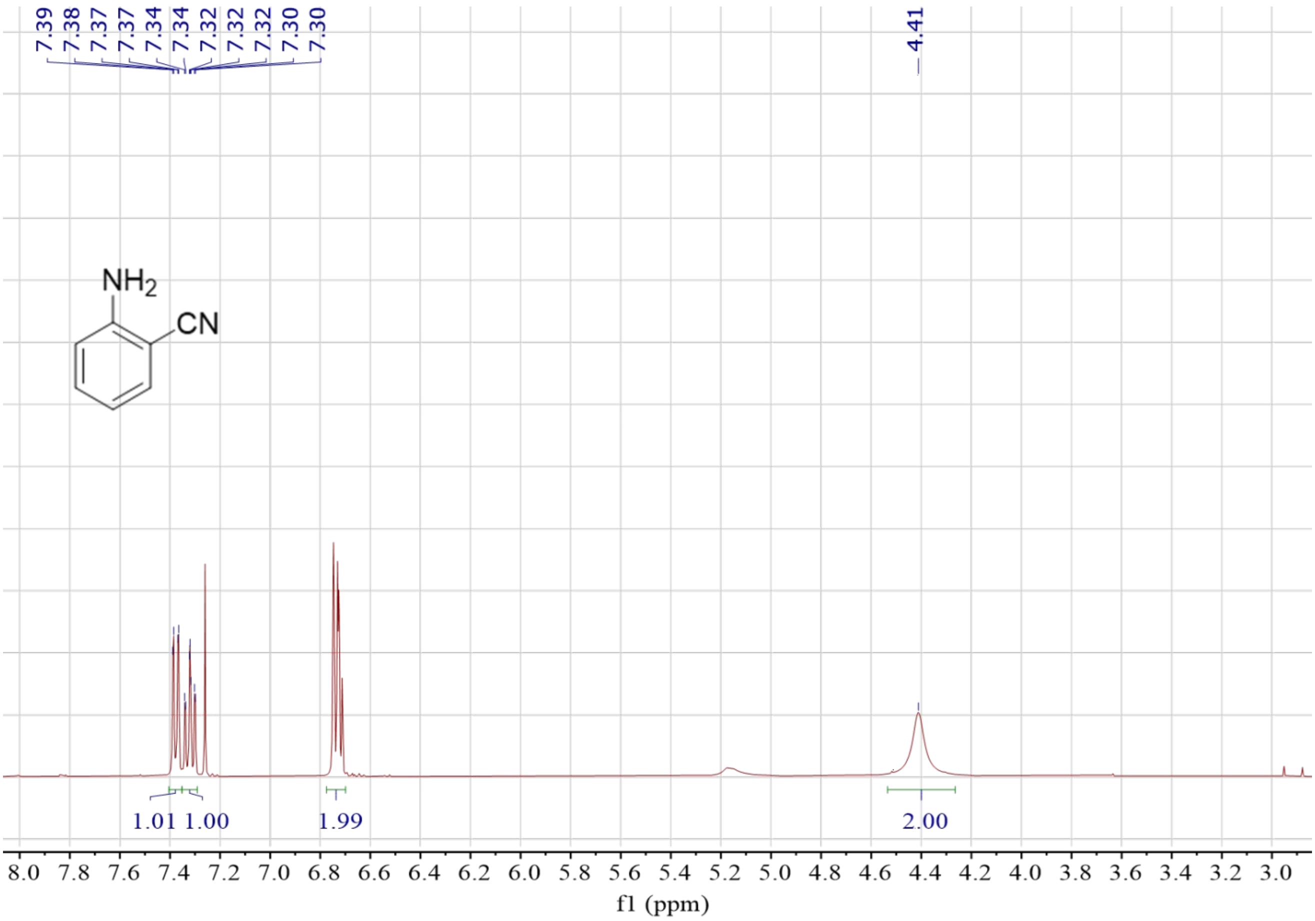

Figure S30: ^{1}H NMR (400 MHz, 298 K) spectrum of 2-aminobenzonitrile (Table 1, entry 4) from the electrocatalytic reaction medium after extraction, concentration, and purification. Spectrum was obtained in $CDCl_3$.

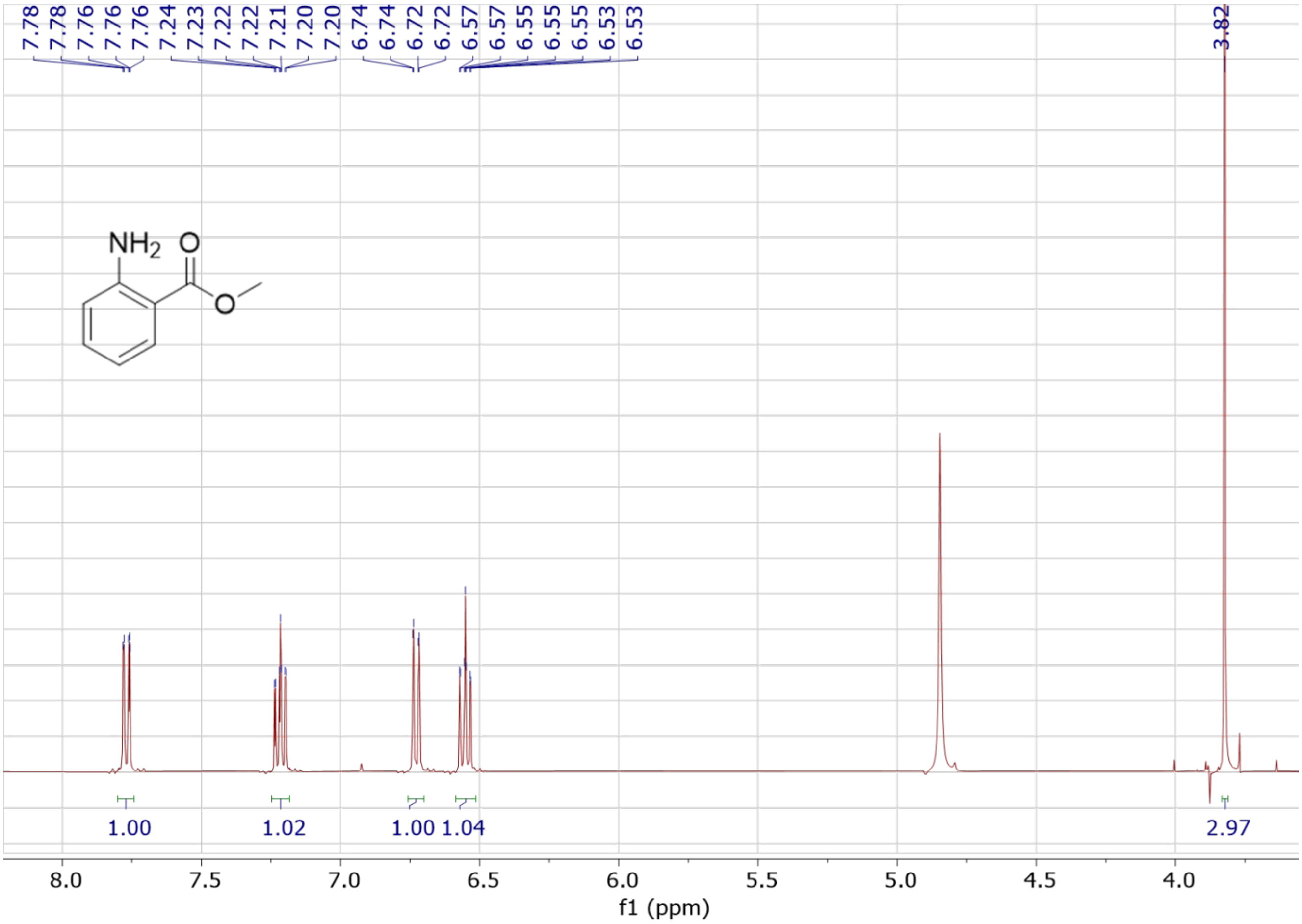

Figure S31: ^{1}H NMR (400 MHz, 298 K) spectrum of methyl-2-aminobenzoate (Table 1, entry 5) from the electrocatalytic reaction medium after extraction and concentration. The spectrum was obtained in MeOD. The peak at 4.8 ppm is due to water in the NMR solvent.

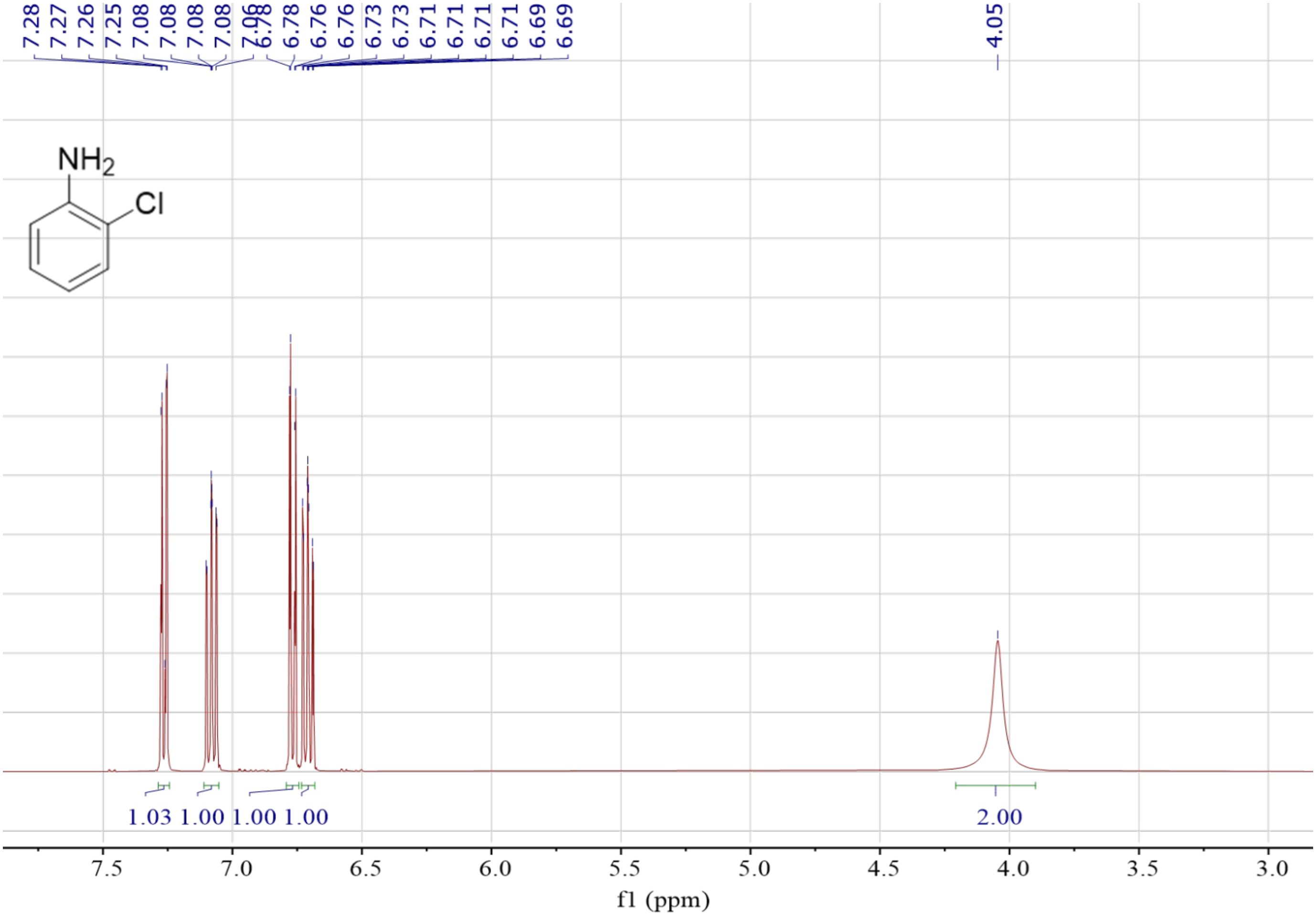

Figure S32: ^{1}H NMR (400 MHz, 298 K) spectrum of 2-chloroaniline (Table 1, entry 6) from the electrocatalytic reaction medium after extraction, concentration, and purification. Spectrum was obtained in $CDCl_3$.

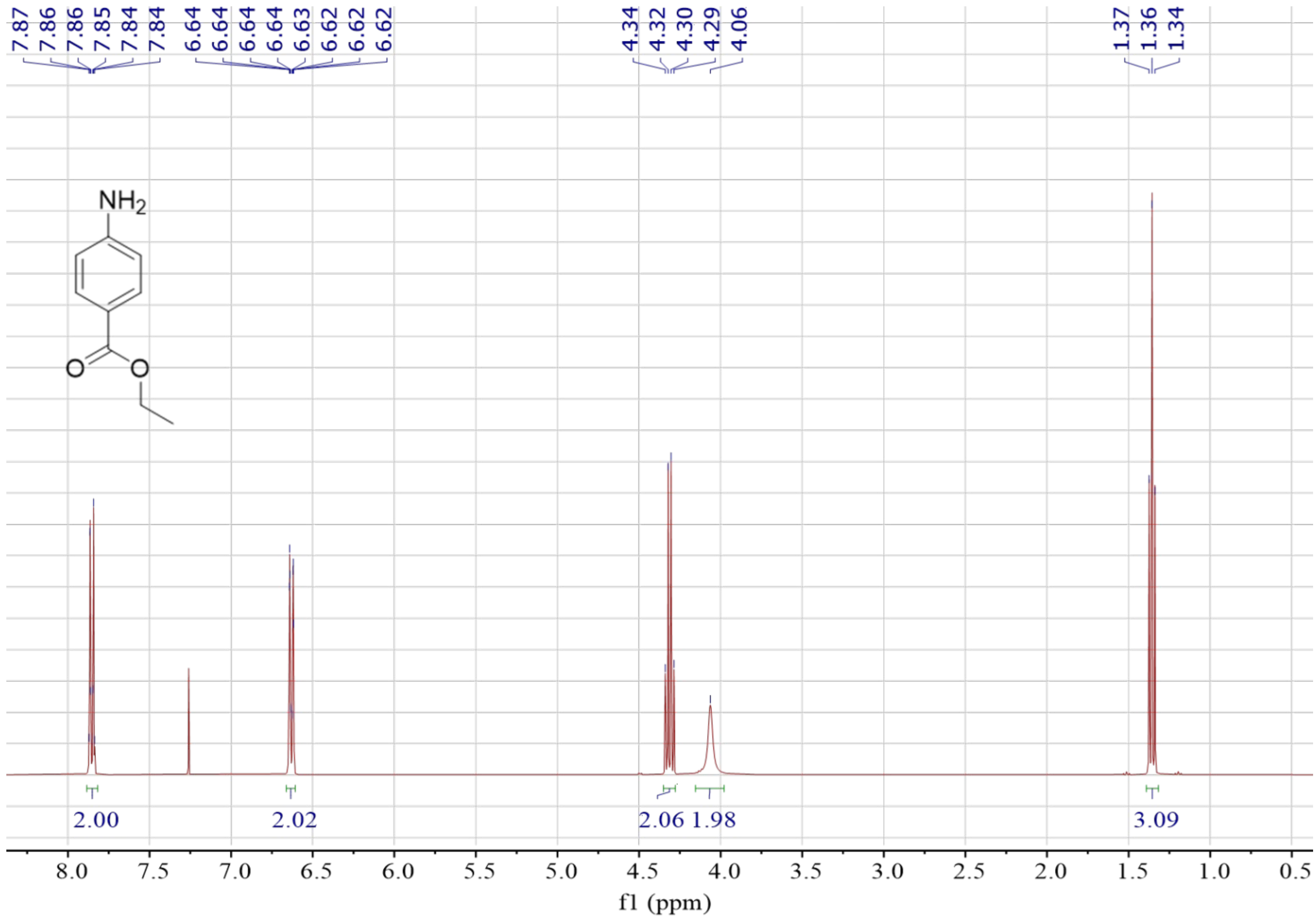

Figure S33: ^{1}H NMR (400 MHz, 298 K) spectrum of ethyl-4-aminobenzoate (Table 1, entry 7) from the electrocatalytic reaction medium after extraction, concentration, and purification. Spectrum was obtained in $CDCl_3$.

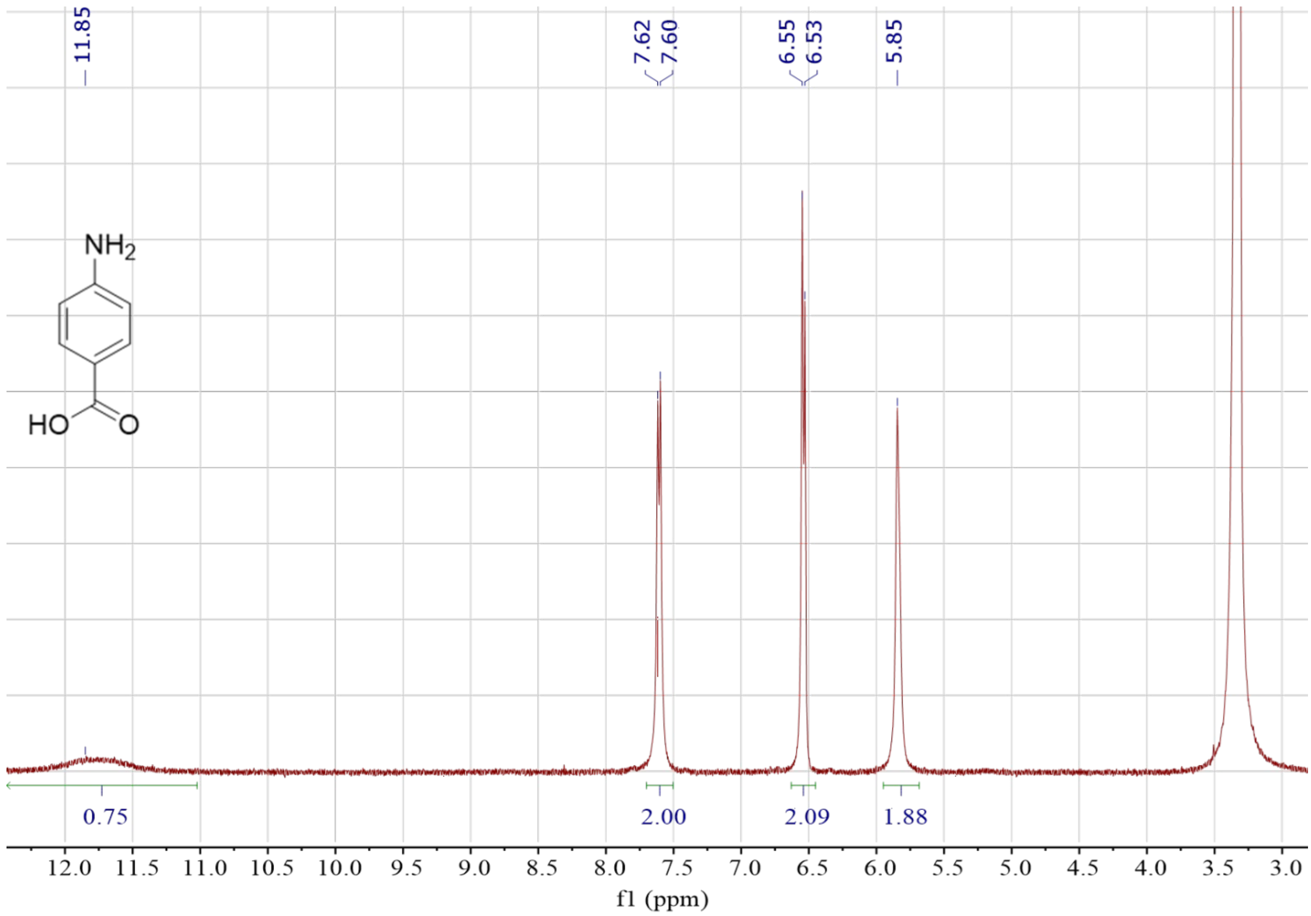

Figure S34: ^{1}H NMR (400 MHz, 298 K) spectrum of 4-aminobenzoic acid (Table 1, entry 8) from the electrocatalytic reaction medium after extraction, concentration, and purification. Spectrum was obtained in d_6-DMSO.

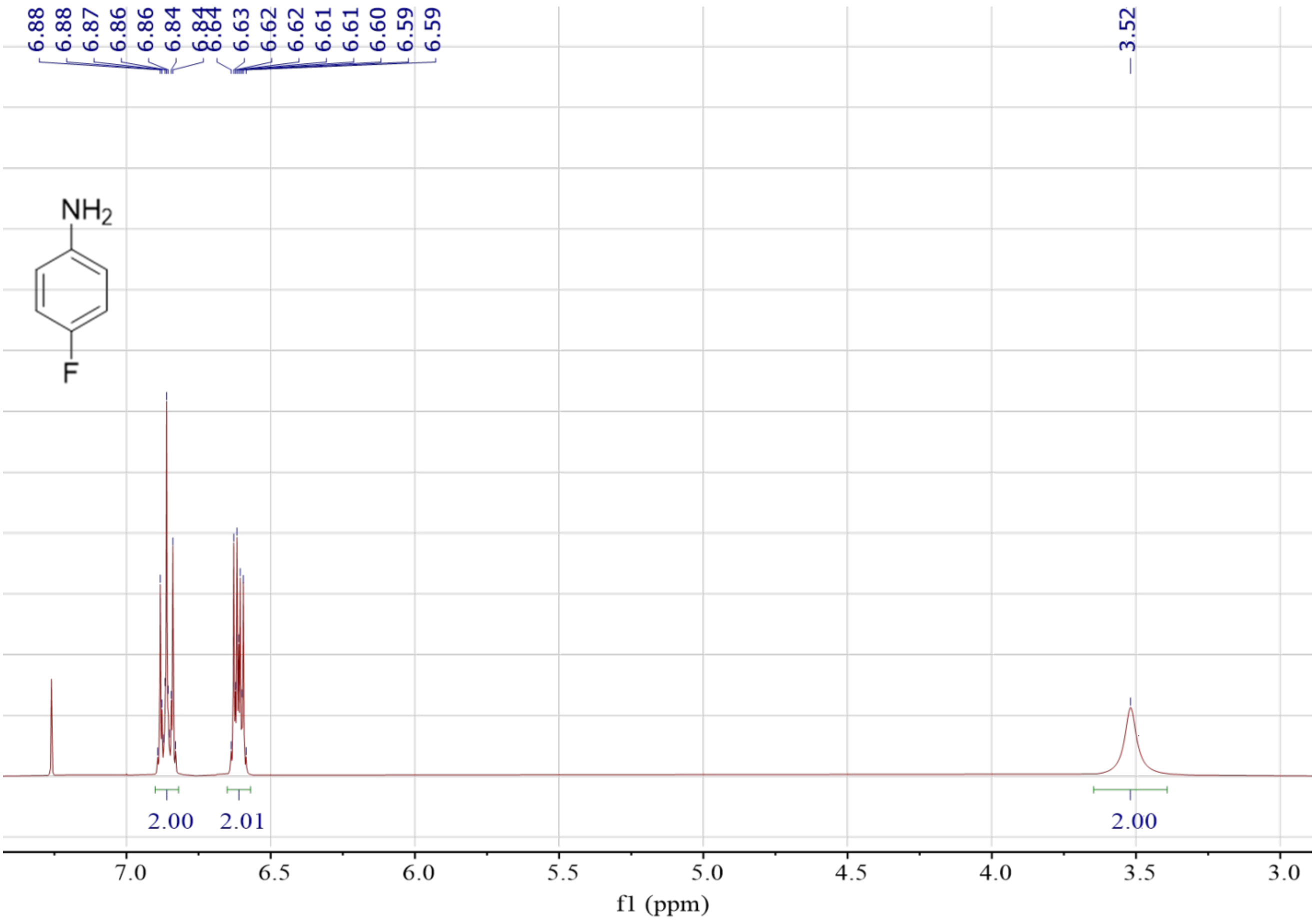

Figure S35: ^{1}H NMR (400 MHz, 298 K) spectrum of 4-fluoroaniline (Table 1, entry 9) from the electrocatalytic reaction medium after extraction, concentration, and purification. Spectrum was obtained in $CDCl_3$.

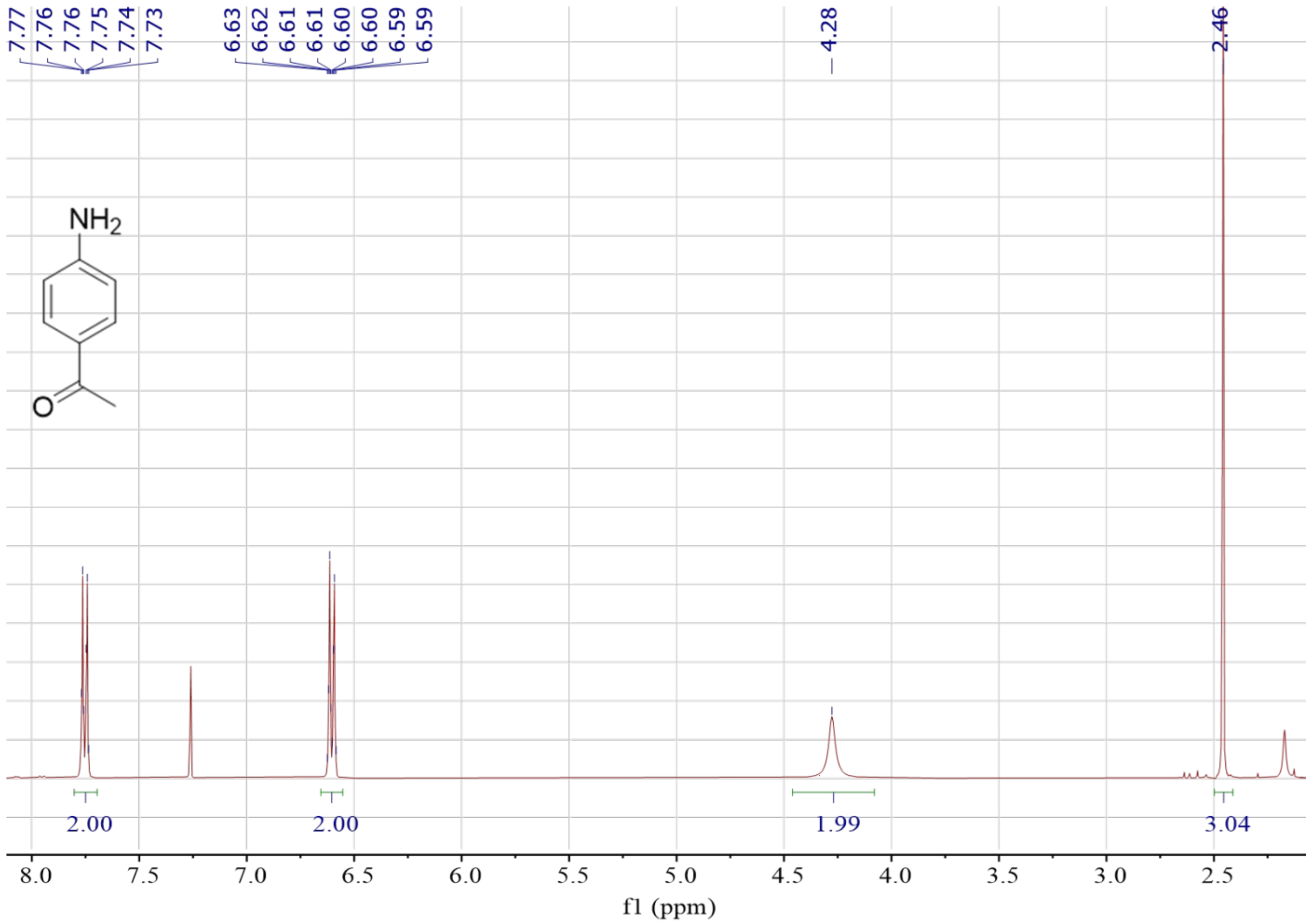

Figure S36: ^{1}H NMR (400 MHz, 298 K) spectrum of 4-aminoacetophenone (Table 1, entry 10) from the electrocatalytic reaction medium after extraction, concentration, and purification. Spectrum was obtained in $CDCl_3$.

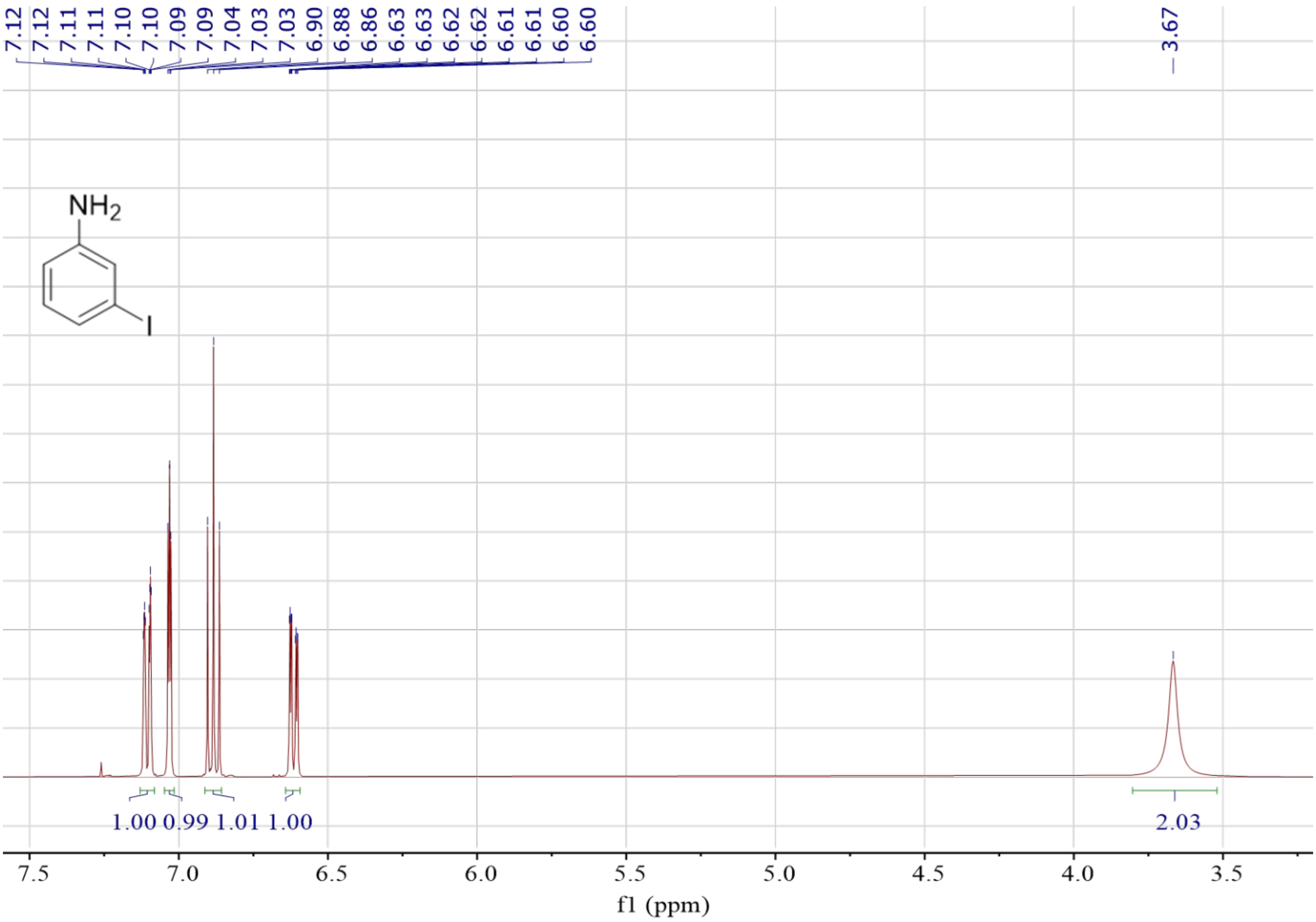

Figure S37: ^{1}H NMR (400 MHz, 298 K) spectrum of 3-iodoaniline (Table 1, entry 11) from the electrocatalytic reaction medium after extraction, concentration, and purification. Spectrum was obtained in $CDCl_3$.

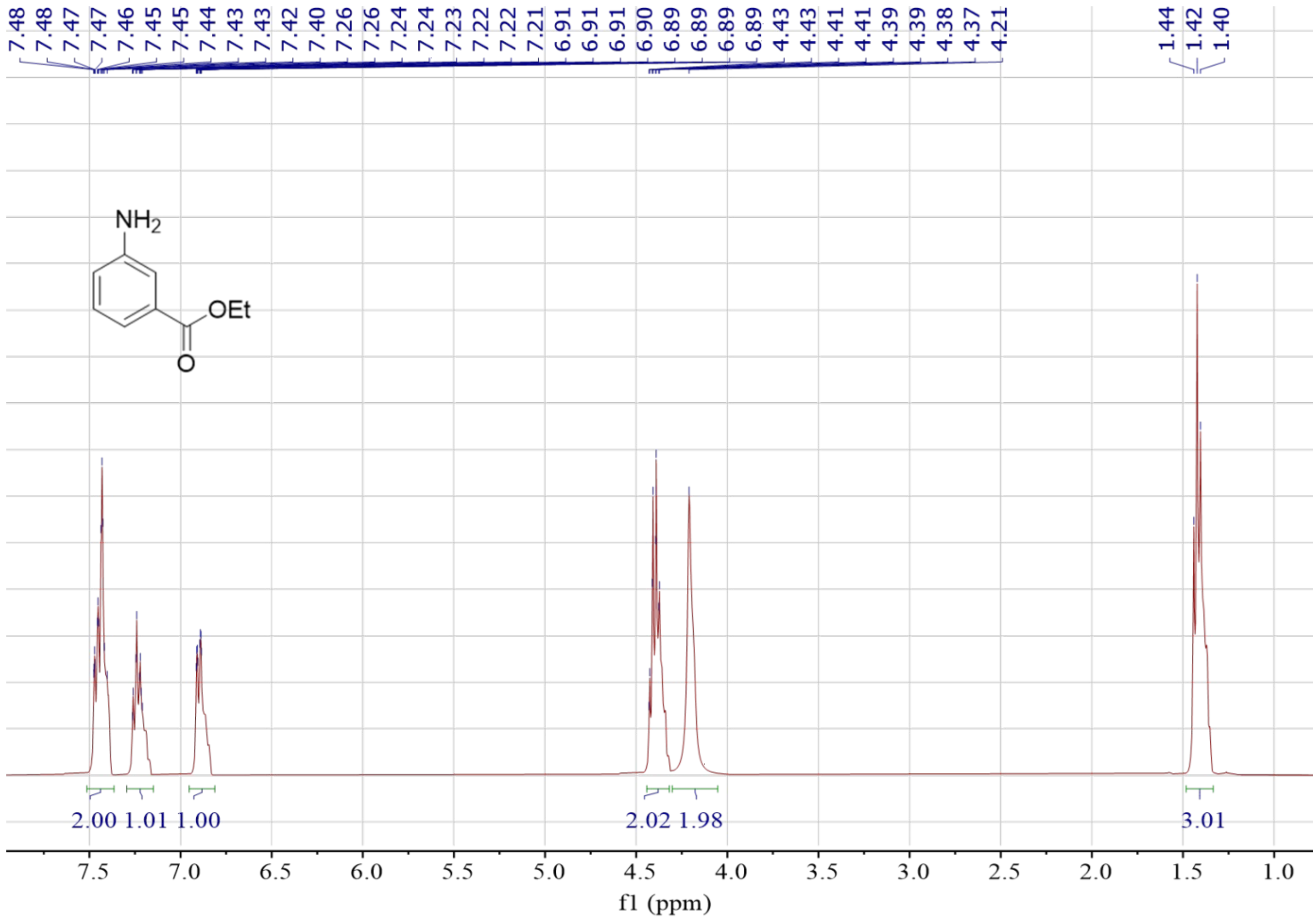

Figure S38: ^{1}H NMR (400 MHz, 298 K) spectrum of ethyl-3-aminobenzoate (Table 1, entry 12) from the electrocatalytic reaction medium after extraction, concentration, and purification. Spectrum was obtained in $CDCl_3$.
